# AMBP protects against aortic valve calcification by inhibiting ERK1/2 and JNK pathways mediated by FHL3

**DOI:** 10.7150/thno.109182

**Published:** 2025-03-18

**Authors:** Chenghu Guo, Xiaoling Liu, Zeyuan Mei, Meiling Chang, Jiaqi Li, Baiqiang Wang, Wei Ji, Meng Zhang, Mei Zhang, Cheng Zhang, Guipeng An

**Affiliations:** 1 State Key Laboratory for Innovation and Transformation of Luobing Theory; Key Laboratory of Cardiovascular Remodeling and Function Research of MOE, NHC, CAMS and Shandong Province; Department of Cardiology, Qilu Hospital of Shandong University, Jinan, China.; 2 Department of Ultrasonography, Affiliated Hospital of Shandong University of Traditional Chinese Medicine, 16369 Jingshi Road, 250014 Jinan, China.

**Keywords:** Alpha-1-microglobulin/bikunin precursor, Calcific aortic valve disease, MAPK pathway, Four-and-a-half LIM domain protein 3, Valvular interstitial cells

## Abstract

**Rationale:** Calcific aortic valve disease (CAVD) is a progressive disorder characterized by aortic valve (AV) calcification and fibrosis. Despite advances in our understanding of CAVD pathogenesis, no drug has proven effective in preventing AV calcification. The aim of this study was to identify the key pathogenic genes in CAVD and elucidate mechanisms that may guide development of new targeted therapies.

**Methods:** A CAVD model was established in ApoE^-/-^ mice by administering a high-cholesterol diet for 24 weeks. An adeno-associated virus was used to induce alpha-1-microglobulin/bikunin precursor (AMBP) overexpression. RNA sequencing, quantitative real-time polymerase chain reaction, western blotting, immunofluorescence, histopathology, and echocardiography were performed to assess AV function. The mechanism of interaction between AMBP and four-and-a-half LIM domain protein 3 (FHL3) was explored using bioinformatics analyses, co-immunoprecipitation, and AlphaFold3-based simulations of crystal structures.

**Results:** RNA sequencing identified AMBP as a key regulator of CAVD. AMBP was increased in calcified AV from CAVD patients and high cholesterol diet (HCD)-induced ApoE^-/-^ mice. *In vivo*, AMBP overexpression significantly reduced HCD-induced AV calcification and fibrosis. *In vitro*, AMBP knockdown elevated osteogenic markers, RUNX2 and OSTERIX, and promoted calcium deposition in valvular interstitial cells induced by osteogenic medium (OM), whereas AMBP overexpression reversed these effects. Mechanistically, AMBP inhibited OM-induced phosphorylation of ERK1/2 (P-ERK1/2) and JNK (P-JNK) by competitively binding to the zinc finger domain of FHL3. This interaction disrupted the protective role of FHL3 in preventing ubiquitin-proteasome-mediated degradation of P-ERK1/2 and P-JNK. P-ERK1/2 and P-JNK inhibitors and agonists confirmed that the protective effects of AMBP against CAVD were mediated via these pathways *in vivo* and *in vitro*.

**Conclusions:** AMBP protects valvular interstitial cells from osteoblastic differentiation and calcium deposit accumulation, thereby alleviating AV calcification. This study sheds additional light on the pathogenesis of CAVD and potential new therapeutic approaches.

## Introduction

As the global population continues to age, the prevalence of CAVD is increasing significantly, making it the most common acquired valvular heart disease 1. Calcified AVS, a late-stage manifestation of CAVD, is characterized by progressive fibrous calcification and AV leaflet thickening, ultimately leading to valve obstruction 2. Within 5 years, approximately 9% of individuals with CAVD develop calcified AVS 3, 4. The morbidity and mortality associated with AVS escalate with the increasing incidence of CAVD, with the prevalence of AVS surpassing 2% among those over 60 years of age and the two-year mortality rate for severe AVS reaching 50% 5, 6.

CAVD is recognized as an active and multifactorial pathogenic process initiated by pathological changes in AV cells, leading to matrix remodeling, subsequent valvular calcification, and hemodynamic obstruction 7. This process is typically divided into two stages: the initial stage, marked by lipid deposition, endothelial injury, dysfunction, and inflammation, and the progressive stage, in which valvular interstitial cells differentiate into myofibroblasts and osteoblast-like cells, activating calcification pathways. These events culminate in end-stage calcification of the aortic valve 8.

With innovative developments in cardiovascular interventional technology, transcatheter active valve replacement, as a representative interventional AV treatment, has been widely promoted and applied globally. However, this treatment method can only be used as a supplementary treatment measure for CAVD, and the service time of artificial valves is limited. Despite advancements in our understanding of the pathophysiological mechanisms of CAVD, no drug therapy has proven effective in preventing or delaying AV calcification. The risk factors for CAVD, such as smoking, hypertension, elevated low-density lipoprotein cholesterol (LDL-C), high lipoprotein(a) levels, and aging, are similar to those associated with atherosclerosis. However, in clinical practice, coronary artery disease and CAVD do not always occur concomitantly and pharmacological treatments for these conditions do not yield similar outcomes. Multiple randomized clinical trials have shown no significant differences between statins and placebos in slowing the progression of AV calcification, suggesting that statins are ineffective during the progressive stages of CAVD 9, 10. This ineffectiveness may be due to the difficulty of reversing valve calcification at this late stage, which implies the existence of lipid-independent mechanisms. Therefore, elucidating the mechanisms underlying CAVD is crucial for developing intervention strategies aimed at preventing or slowing CAVD progression.

In this study, we performed RNA-seq to identify the key pathogenic genes in CAVD. We aimed to investigate the regulatory role of AMBP in CAVD progression and the underlying molecular mechanisms.

## Methods

Expanded methods are available in the Supplemental Material.

### Patients and sample acquisition

We included patients (aged 50-75 years) who underwent AV replacement for severe AVS and collected calcified AV tissue. Non-calcified AV tissues from patients (aged 50-75 years) who underwent heart transplantation (recipient heart) or AV resection due to aortic dissection were collected as controls (non-CAVD). The exclusion criteria included: rheumatic heart disease, moderate or severe aortic insufficiency, chronic kidney disease with estimated glomerular filtration rate ≤ 30 mL/min/1.73 m^2^, intestinal diseases (Crohn's disease and inflammatory bowel disease), autoimmune diseases, treatment with antibiotics or probiotics within 1 month due to infection, or lack of clinical data. Demographic characteristics, medical history, physical examination results, major laboratory test results, and cardiac imaging data were collected from all patients. The study complied with the principles of the Declaration of Helsinki and was approved by the Research Ethics Committee of Qilu Hospital of Shandong University (ethics approval number: KYLL-202208-003-1). Written informed consent was obtained from all patients.

### RNA-seq data generation and bioinformatics analysis

Total RNA was extracted using TRIzol reagent (Invitrogen, Carlsbad, CA, USA), and its integrity was assessed using an Agilent Bioanalyzer 2100 (Agilent Technologies, Santa Clara, CA, USA). RNA concentration and purity were measured using a Qubit® 3.0 Fluorometer (Life Technologies, Carlsbad, CA, USA) and a Nanodrop One spectrophotometer (Thermo Fisher Scientific Inc., Waltham, MA, USA). Paired-end libraries were prepared using the Stranded mRNA-seq Lib Prep Kit for Illumina (ABclonal, Hubei, China) and sequenced using Illumina NovaSeq 6000 (Illumina, San Diego, CA, USA).

For bioinformatic analysis, sequencing data were processed using Fastp to filter out low-quality reads and adaptors 11. Clean reads were aligned to the human reference genome (GRCh38.102) using Hisat2 (version 2.0.5) 12, and the resulting SAM files were converted to the BAM format and sorted using SAMtools (version 1.3.1). Differentially expressed genes (DEGs) were identified using StringTie for fragment counting (https://ccb.jhu.edu/software/stringtie/), TMM for normalization 13, and EdgeR for differential expression analysis 14. Visualization was performed using the heatmap and ggplot2 packages in R (version 3.2.5, Vienna, Austria), with DEGs defined as |log2(FC)| > 1 and q-value (FDR-adjusted P-value) < 0.05. The DEGs were further analyzed by Gene Ontology (GO) and Kyoto Encyclopedia fo Genes and Genomes (KEGG) enrichment using Metascape 15. Protein-protein interaction (PPI) networks were constructed using STRING 16 and visualized using Cytoscape (version 3.9.1) 17 with hub genes identified via CytoHubba 18. The RNA-seq data are available under the NCBI Gene Expression Omnibus (GEO) accession number GSE235995.

### Total RNA isolation in tissue and cells and qRT-PCR

Total RNA from the aortic valvular tissue and cells was isolated using the TRIzol reagent (Invitrogen) and reverse-transcribed using the PrimeScript RT Reagent Kit (Takara Biomedical Technology, Beijing, China). qRT-PCR amplification was performed using SYBR PCR mix (Roche, Mannheim, Germany) with specific primers (Table S1) in a Bio-Rad CFX96TM Real-Time PCR detection system (Bio-Rad Laboratories, Inc., Hercules, CA, USA).

### Isolation of primary VICs, cell culture, and transfection

Primary human VICs were isolated from the AVs of patients without CAVD who underwent heart transplantation (recipient heart) or AV resection due to aortic dissection, as previously reported 19. The clinical characteristics of the patients used for cell isolation are listed in Table S2. materials. VICs were cultured in Dulbecco's modified Eagle's medium (DMEM) containing 10% fetal bovine serum (FBS) and 1% penicillin and streptomycin in a humidified atmosphere with 5% CO_2_ at 37 °C. Osteogenic medium (OM) was used to stimulate osteogenic differentiation 20. VICs were transfected with small interfering RNAs (siRNAs) targeting* AMBP* and *FHL3* using the Lipofectamine RNAi MAX Transfection Reagent (Cat. No. 13778150; Thermo Fisher Scientific) or *AMBP* overexpressing adenovirus (BIOSUN, Jinan, China). For mechanistic experiments, VICs were pretreated with a P-ERK1/2 inhibitor (50 µM; Cat. No. PD98059; TOPSCIENCE, Shanghai, China), P-JNK inhibitor (10 µM; Cat. No. SP600125; TOPSCIENCE), or proteasome inhibitor MG132 (10 µM; Cat. No. HY-13259; MedChemExpress, Shanghai, China) for 1 h, and then transfected with siRNA or adenovirus. For the PPI experiments, HEK293T cells were transfected with plasmids encoding C-terminal FLAG-tagged *AMBP* and C-terminal HA-tagged *FHL3* full-length/truncated mutants using Lipofectamine 3000 (Cat. No. L3000015; Thermo Fisher Scientific) following the manufacturer's protocol.

### Protein extraction and western blot analysis

VICs were lysed in RIPA buffer (Sigma-Aldrich, St Louis, MO, USA) with 1X protease inhibitor cocktail (Cat. No. 04693132001; Roche, Indianapolis, IN, USA). The extracted proteins were separated using 4-10% gradient Bis-Tris SDS-gels (Bio-Rad, Hercules, CA, USA) and then transferred to nitrocellulose membranes (Millipore, Billerica, MA, USA). The blots were incubated with 5% non-fat milk at room temperature (23-27 °C) for 1 h and then with primary antibodies at 4 °C overnight. The next day, the membranes were incubated with horseradish peroxidase (HRP)-conjugated secondary antibodies (Cat. No. ab6721 for Rabbit; ab6728 for Mouse; Abcam, Cambridge, MA, USA) at room temperature (23-27 °C) for 1 h and were visualized using an ECL western blotting detection kit (Millipore, Temecula, CA, USA). The intensities of the bands were quantified using ImageJ software (National Institutes of Health, Bethesda, MD, USA). Protein expression levels were normalized to those of GAPDH or beta-Actin.

### Alizarin Red S staining of VICs

The treated primary VICs were washed three times for 3 min with phosphate-buffered saline and fixed for 30 min in 4% paraformaldehyde, followed by incubation with 0.2% Alizarin Red solution (Cat. No. C0148S; Servicebio, Wuhan, China) for 1 h. Double-distilled water was used to remove the excess dye. Alizarin Red S staining was observed and photographed using an inverted microscope (Olympus, Tokyo, Japan). Representative images from each group were randomly selected.

### Co-immunoprecipitation

Forty-eight h after plasmid transfection, cells were harvested and lysed in non-denaturing lysis buffer (Cat. No. P0013; Beyotime Biotechnology) for 30 min. Protein A/G magnetic beads (Cat. No. HYK0202; MedChemExpress) were incubated with IP-grade antibodies at room temperature for 1 h, followed by the addition of the cell lysate, which was then incubated overnight at 4 °C with rotation. The next day, the beads were collected, and the supernatant was discarded. After four washes with PBS containing 0.5% Tween-20, SDS-PAGE loading buffer was added, and the samples were heated at 95 °C for 5 min. The collected supernatants were analyzed by western blotting.

### Immunofluorescence staining

Human AV tissue sections were dewaxed, antigen-repaired, and treated with 0.1% TritonX-100 for 10 min. Sections were then incubated with 2.5% normal goat serum (Cat. No. G1208; Servicebio) for 30 min at room temperature and then incubated with primary antibodies against AMBP (Cat. No. ER1803-35; HUABIO, Hangzhou, China) and VIM (Cat. No. 5741; Cell Signaling Technology, Danvers, MA, USA) overnight at 4 °C. The following day, the sections were incubated with Alexa Fluor 594 (Cat. No. ab150120; Abcam) and 488 (Cat. No. ab150081; Abcam) secondary antibodies (1:200) for 1 h at 37 °C in the dark. Nuclei were stained with DAPI (Cat. No. ab104139; Abcam). Immunofluorescent staining was performed utilizing a fluorescence microscope (Olympus).

### Animal experiments

All animal procedures were approved by the Research Ethics Committee of Qilu Hospital of Shandong University (approval number: KYLL-2023(ZM)-360) and adhered to Directive 2010/63/EU for the protection of animals used for scientific purposes. Six-week-old male ApoE^-/-^ mice (C57BL/6J background) were obtained from GemPharmatech Co., Ltd. (Jiangsu, China) and maintained in a pathogen-free, temperature-controlled environment with a 12:12-h light-dark cycle. The mice received tail vein injections of adeno-associated virus (AAV) subtype 2 21-23 overexpressing AMBP (AAV2-*Ambp*, 2×10^11^ vg/mouse; CMV promoter; GenePharma, Shanghai, China) or negative control (AAV2-NC, 2×10^11^ vg/mouse; CMV promoter; GenePharma, Shanghai, China ) for 2 weeks, followed by a 0.2% high-cholesterol diet (HCD) for 24 weeks to induce AV calcification 19, 24. For *in vivo* mechanistic experiments, mice received AAV2-*Ambp* injection for 2 weeks and were pre-treated with the ERK activator, bortezomib (Cat. No. S1013; Selleck, Shanghai, China) and the JNK activator, anisomycin (Cat. No. S7409; Selleck) before initiating the HCD. At the end of experiments for all animals, echocardiography was performed using an 18-38-MHz phased array transducer (MS400) and the VisualSonic VeVo 2100 Imaging System (Toronto, Canada) to measure the transvalvular peak jet velocity and AV peak pressure. Mice were anesthetized with 2% isoflurane and placed on a heated platform at 37 ± 1 °C. Upon completion of imaging, the mice were euthanized with a lethal dose of sodium pentobarbital (100 mg/kg), and AV tissues and blood were collected for analysis. Histological examinations were conducted on OCT-embedded frozen sections (4 µm) using hematoxylin and eosin (HE), Masson's trichrome, Alizarin Red S, and Von Kossa staining. The stained sections were photographed under a light microscope (Olympus).

### Molecular modeling and docking

The AMBP and FHL3 protein sequences were retrieved from the UniProt database. The sequences were submitted to the AlphaFold server for protein structure prediction and interaction analysis 25. The predicted interaction models were visualized using PyMOL (PyMOL Molecular Graphics System, version 3.0.4, Schrodinger, New York, United States), in which the polar interactions between proteins were highlighted to illustrate the key binding sites.

### Statistical analysis

Data were analyzed using GraphPad Prism 9 software (San Diego, CA, USA). Continuous variables were expressed as mean ± standard error of the mean (SEM) when conforming to a normal distribution; otherwise, they were expressed as median and interquartile range. Normality tests were performed using the Shapiro-Wilk normality test. For normally distributed data, an unpaired two-tailed Student's t-test was used to determine statistically significant differences between two groups. One-way analysis of variance followed by the Bonferroni multiple comparison test (with a mixed model with different numbers of replicates per condition) was performed to determine the statistical difference between multiple groups with one variable and a normal distribution. Two-way analysis of variance followed by the Bonferroni multiple comparison test was used to compare multiple groups with more than one variable. For non-normally distributed data, a nonparametric statistical Kruskal-Wallis test followed by Dunn's post hoc test was performed for multiple comparisons. The biological and technical replicates are explicitly specified in the respective figure legends to ensure reproducibility and statistical robustness. Statistical significance was set at p < 0.05.

## Results

### AMBP is increased in calcified AVs from patients with CAVD and in mice

Calcified human AV tissue was collected from five patients (four men) with severe AVS who underwent aortic valvular replacement. Control AV tissue (non-CAVD) was collected from one patient (female) who underwent heart transplantation for refractory dilated cardiomyopathy (recipient heart) and three patients (male) who underwent AV resection for aortic dissection. Patients in the non-CAVD group were younger than those in the CAVD group (51.75 ± 4.64 years *vs.* 64.80 ± 1.69 years, p < 0.05). No significant differences in other demographic characteristics, history of non-cardiac disease, routine laboratory indicators, or cardiac function were observed between the two groups (Table S3).

**Figure 1:**
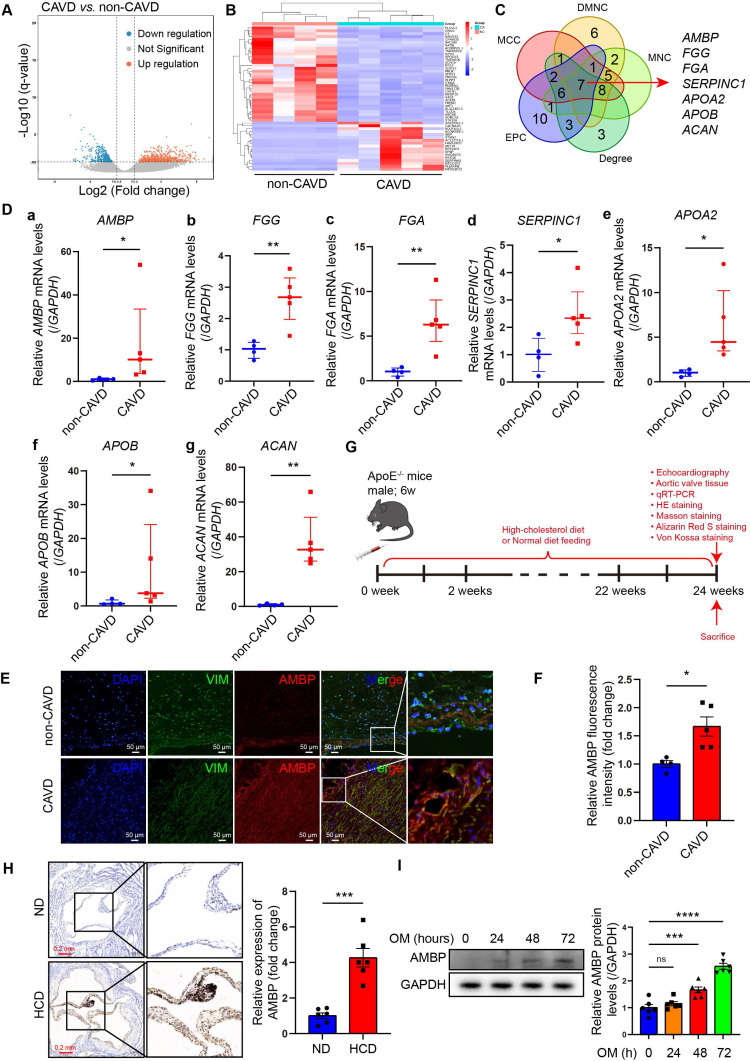
** AMBP increases in calcified AVs from CAVD patients and ApoE^-/-^ mice.** (**A**) Volcano plot of DEGs. Blue, red, and grey dots represent downregulated genes, upregulated genes, and genes without significant expression changes in CAVD versus non-CAVD groups. Screening parameters were |log2(FC)| > 1 and q-value (FDR adjusted p-value) < 0.05. (**B**) Heatmap of top 50 DEGs; red and blue represent high and low expression, respectively. **(C)** Venn diagram verifying overlap of hub genes from five algorithms. (**D**) qRT-PCR examining the mRNA expression levels of key hub genes: **(a)** *AMBP*, **(b)** *FGG*, **(c)** *FGA*, **(d)** *SERPINC1*, **(e)** *APOA2*, **(f)** *APOB*, and **(g)** *ACAN* in AV tissue obtained from CAVD (n = 5) and non-CAVD (n = 4) groups. (**E-F**) Representative immunofluorescence images (**E**) and quantitative analysis (**F**) of AMBP expression (red) in VICs (green) of AV tissue obtained from CAVD (n = 5) and non-CAVD (n = 4) groups (scale bar = 50 µm). (**G**) Schematic representation of CAVD modeling in ApoE^-/-^ mice. (**H**) Representative immunohistochemical images (scale bar = 0.2 mm) and quantitative analysis of AMBP expression in AV tissues from HCD and ND mice (n = 6). (**I**) Representative western blots images and quantitative analysis of temporal expression pattern of AMBP in VICs under OM induction (n = 6). Values are presented as median with interquartile range (D) or mean ± SEM (F, H, and I). *p < 0.05; **p < 0.01; ***p < 0.001; ****p < 0.0001; ns, non-significant. Abbreviations: AMBP, alpha-1-microglobulin/bikunin precursor; AV, aortic valve; CAVD, calcific aortic valve disease; DEG, differentially expressed gene; FC, fold change; FDR, false discovery rate; HCD, high-cholesterol diet; ND, normal-diet; OM, osteogenic medium; qRT-PCR, quantitative reverse transcription polymerase chain reaction; SEM, standard error of the mean; VICs, valvular interstitial cells.

RNA-seq was performed on AVs from the CAVD and non-CAVD groups to identify DEGs. In total, 870 DEGs were identified, with 525 upregulated and 345 downregulated genes (|log2(FC)| > 1 and q < 0.05) (Figure 1A-B). The top 50 DEGs are listed in Table S4. GO enrichment analysis revealed that DEGs were enriched in 582 GO terms (q ≤ 0.05), including 480 biological processes (BP), 60 cellular components (CC), and 42 molecular functions (MF). The top 30 GO terms were primarily related to lipid composition, metabolism, and immune cell processes (Figure S1A). KEGG pathway analysis indicated that the DEGs were associated with extracellular matrix (ECM) interaction, mineral absorption, and cholesterol metabolism (Figure S1B). To explore DEG interactions, a PPI network was constructed using the STRING database, consisting of 229 nodes and 328 edges with an interaction threshold score of 0.9 (Figure S2A). This network included 146 upregulated and 79 downregulated genes in the CAVD group. Module analysis using Molecular Complex Detection identified 17 significant modules (Table S5), and the top seven modules are displayed in Figure S2B. Five algorithms (Degree, DMNC, EPC, MCC, and MNC) were applied using the CytoHubba plug-in to identify key hub genes. The top 30 hub genes were identified (Table S6), and a Venn diagram identifed seven overlapping hub genes: *AMBP*, *FGG*, *FGA*, *SERPINC1*, *APOA2*, *APOB*, and *ACAN* (Figure 1C). The expression of these seven key hub genes in AV samples was further validated by qRT-PCR. Consistent with the RNA-seq data, all seven genes were significantly upregulated in the CAVD group compared to those in the non-CAVD group, indicating their potential role in CAVD pathogenesis (Figure 1D).

According to the functional analysis of genes and literature searches, *ACAN*, *FGA*, *FGG*, and *SERPINC1* are mainly involved in the expression or degradation of encoded ECM proteins, which are primarily involved in the final stage of AV calcification. *APOA2* and *APOB* encode apolipoproteins A2 and B, respectively, and participate in lipid metabolism, which is involved in the initial stages of CAVD pathogenesis. Unlike others, the role of AMBP, the plasma glycoprotein α-1-microglobulin (A1M) and bikunin precursor protein, in the pathogenesis of CAVD had yet to be clarified.

The *AMBP* gene encodes plasma glycoprotein A1M and bikunin precursor protein. AMBP is hydrolyzed into two proteins with different functions: A1M and bikunin. A1M is an antioxidant and tissue-cleaning protein with reductase, heme, and free radical binding properties and plays a role in regulating inflammation. However, bikunin is a structural component of the ECM. Therefore, we speculated that AMBP may be an important regulatory factor involved in the occurrence and development of CAVD.

Immunofluorescence staining revealed that AMBP co-localized with vimentin, a marker protein specific to VICs, in AV tissues from patients with CAVD (Figure 1E). These findings strongly suggest that VICs may be the primary cellular source of elevated AMBP expression in calcified AV tissue. Quantitative analysis of immunofluorescence staining revealed that AMBP expression was significantly higher in AV tissues from patients with CAVD compared with those from patients without CAVD (Figure 1F). To further explore the role of AMBP, we developed a CAVD model in ApoE^-/-^ mice by utilizing a HCD for 24 weeks to induce AV calcification (Figure 1G) 19, 24. Immunohistochemical analysis revealed a significant upregulation of AMBP expression in AV tissues from HCD mice compared with that in normal diet (ND) mice (Figure 1H). We investigated the temporal expression patterns of AMBP in VICs induced by OM *in vitro*. As shown in Figure S3, treatment with OM induced a time-dependent upregulation of osteoblastic differentiation markers RUNX2 and OSTERIX. Notably, AMBP expression exhibited a similar time-dependent pattern, with significant upregulation observed at 48 and 72 hours compared to the baseline (0 hours) control group (Figure 1I). These findings indicate that AMBP is upregulated in calcified AV *in vivo* and in osteogenic-induced VICs *in vitro*.

### AMBP overexpression mitigates AV calcification induced by a HCD in ApoE^-/-^ mice

**Figure 2:**
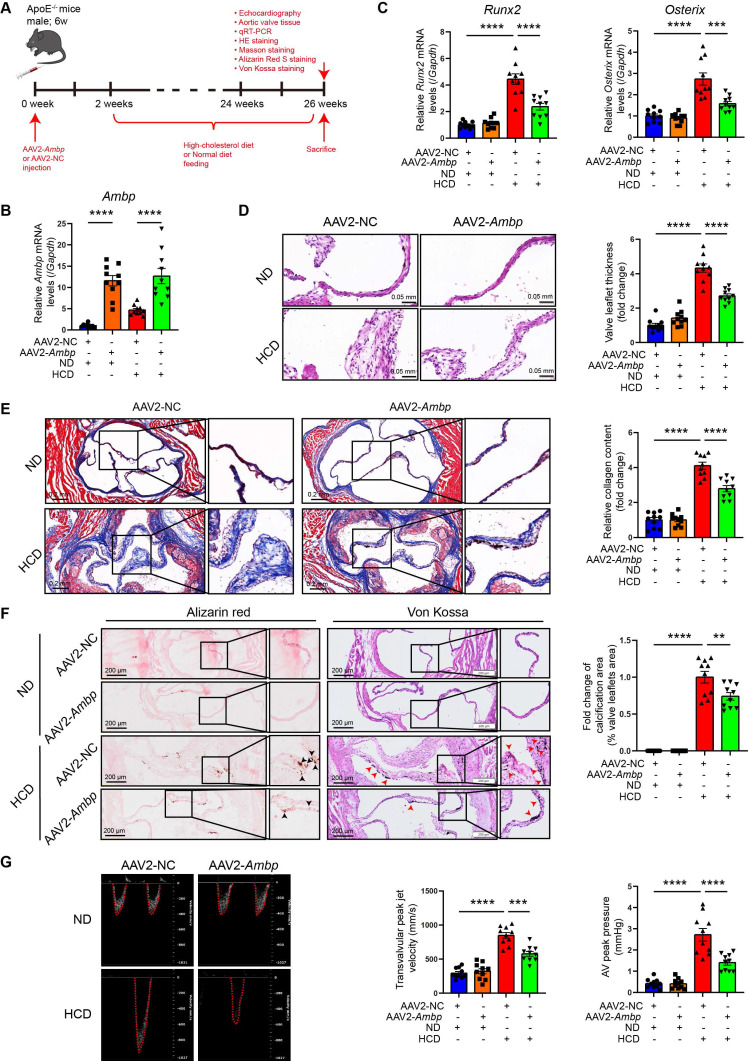
** AMBP overexpression mitigates AV calcification induced by HCD in ApoE^-/-^ mice.** (**A**) Schematic diagram depicting the time course of HCD-induced AV calcification in ApoE^-/-^ mice. (**B-C**) qRT-PCR was used to quantify the mRNA expression levels of *Ambp* **(B)**, *Runx2*, and *Osterix* (**C**) in AV tissue from ApoE^-/-^ mice (n = 10). (**D**) Representative HE images and quantitative analysis of AV thickening in ApoE^-/-^ mice (n = 10, scale bar = 0.05 mm). (**E**) Representative Masson staining images and quantitative analysis of AV fibrosis in ApoE^-/-^ mice (n = 10, scale bar = 0.2 mm). (**F**) Representative Alizarin Red S (black arrows indicate calcified lesions) and Von Kossa (red arrows indicate calcified lesions) staining images and quantitative analysis of AV calcification in ApoE^-/-^ mice (n = 10, scale bar = 200 µm applies to the left wide-field images). (**G**) Representative images of pulsed Doppler flow spectrum at AV and quantitative analysis of transvalvular peak jet velocity and AV peak pressure with echocardiography in ApoE^-/-^ mice (n = 10). Values are presented as mean ± SEM. *p < 0.05; **p < 0.01; ***p < 0.001; ****p < 0.0001. Abbreviations: AMBP, alpha-1-microglobulin/bikunin precursor; AAV2-NC, negative control adeno-associated virus subtype 2; AAV2-*Ambp*, *Ambp* overexpressing adeno-associated virus subtype 2; AV, aortic valve; HE, hematoxylin eosin; HCD, high-cholesterol diet; qRT-PCR, quantitative reverse transcription polymerase chain reaction; SEM, standard error of the mean.

To explore the effect and therapeutic potential of AMBP overexpression in an animal model of AV calcification, 6-week-old ApoE^-/-^ mice were injected with an AAV2 overexpressing *Ambp* for 2 weeks. Subsequently, mice were fed a HCD for 24 weeks to induce AV calcification (Figure 2A). Two weeks after AAV2 injection, the efficiency of AMBP overexpression in the AV was verified by qRT-PCR (Figure 2B) and immunohistochemical staining (Figure S4). At the end of the modeling period, qRT-PCR analysis of osteoblastic differentiation marker genes in AV leaflets revealed that *Ambp* overexpression significantly inhibited the HCD-induced expression of *Runx2* and *Osterix* (Figure 2C). HE staining indicated that *Ambp* overexpression significantly inhibited AV leaflet thickness induced by HCD (Figure 2D). Masson's trichrome staining showed that *Ambp* overexpression significantly inhibited HCD-induced leaflet fibrosis (Figure 2E). Alizarin Red S and Von Kossa staining demonstrated *Ambp* overexpression significantly inhibited HCD-induced calcification of AV leaflets (Figure 2F). Echocardiographic assessment revealed that *Ambp* overexpression significantly reduced the transvalvular peak jet velocity and AV peak pressure in HCD-fed ApoE^-/-^ mice (Figure 2G). Furthermore, no differences in serum glucose and lipid levels, including total cholesterol, triglycerides, high-density lipoprotein cholesterol, and LDL-C, were observed between AAV2-NC and AAV2-*Ambp* mice fed the HCD, suggesting that the protective effects of AMBP on CAVD are independent of glucose and lipid metabolism (Figure S5). Taken together, *Ambp* overexpression mitigates AV calcification induced by a HCD in ApoE^-/-^ mice.

### AMBP protects against osteoblastic differentiation and calcification of VICs *in vitro*

**Figure 3:**
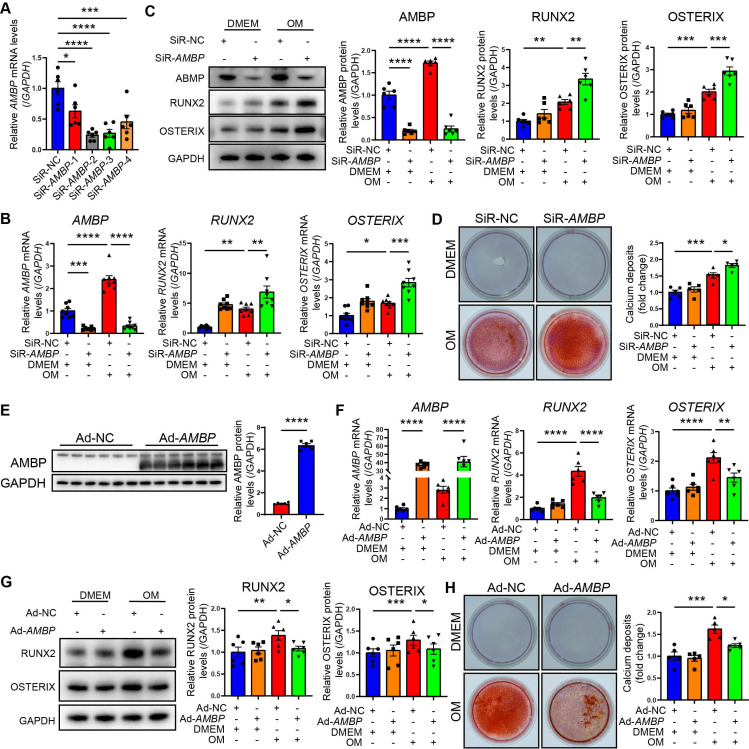
** AMBP protects against VICs osteoblastic differentiation and calcification *in vitro*.** (**A**) Four siRNAs specifically targeting *AMBP* were designed and transfected into VICs. qRT-PCR evaluates efficiency of *AMBP* knockdown (n = 6). (**B**) *AMBP* knockdown significantly enhances OM-induced *RUNX2* and *OSTERIX* expression at mRNA levels, as demonstrated by qRT-PCR (n = 8). VICs are transfected with SiR-NC or SiR-*AMBP* for 24 h and then cultured in DMEM or OM for 72 h. (**C**) *AMBP* knockdown significantly enhances OM-induced RUNX2 and OSTERIX expression at protein levels, as demonstrated by Western blot analysis (n = 6). VICs are transfected with siR-NC or SiR-*AMBP* for 24 h and then cultured in DMEM or OM for 72 h. (**D**) Alizarin Red S staining demonstrating that *AMBP* knockdown significantly enhances OM-induced accumulation of calcium deposits and formation of calcified nodules (n = 5). VICs are transfected with SiR-NC or SiR-*AMBP* for 24 h and then cultured in DMEM or OM for 7 d. (**E**) Representative Western blot images and quantitative analysis of the overexpression efficacy of AMBP in VICs transfected with Ad-*AMBP* (n = 6). (**F**) *AMBP* overexpression significantly inhibits OM-induced *RUNX2* and *OSTERIX* expression at mRNA levels, as demonstrated by qRT-PCR (n = 6). VICs are transfected with Ad-NC or Ad-*AMBP* for 24 h and then cultured in DMEM or OM for 72 h. (**G**) *AMBP* overexpression significantly inhibits OM-induced RUNX2 and OSTERIX expression at protein levels, as demonstrated by Western blot analysis (n = 6). VICs are transfected with Ad-NC or Ad-*AMBP* for 24 h and then cultured in DMEM or OM for 72 h. (**H**) Alizarin Red S staining shows that *AMBP* overexpression protects against the OM-induced accumulation of calcium deposits and formation of calcified nodules (n = 5). VICs are transfected with Ad-NC or Ad-*AMBP* for 24 h and then cultured in DMEM or OM for 7 d. Values are presented as mean ± SEM. *p < 0.05; **p < 0.01; ***p < 0.001; ****p < 0.0001. Abbreviations: Ad-NC, negative control adenovirus; Ad-*AMBP*, *AMBP* overexpressing adenovirus; DMEM, Dulbecco's modified Eagle's medium; OM, osteogenic medium; qRT-PCR, quantitative reverse transcription polymerase chain reaction; SiR-NC, small interfering RNA negative control; SiR-*AMBP*-1, -2, -3, and -4, No.1, No.2, No.3, and No.4 small interfering RNA targeting* AMBP*; VICs, valvular interstitial cells.

To investigate the effect of AMBP on VICs calcification *in vitro*, we investigated four siRNAs targeting *AMBP* for knockdown. The second siRNA demonstrated the highest knockdown efficiency in primary VICs, significantly reducing *AMBP* expression, and validating its use in further functional studies (Figure 3A and Figure S6). qRT-PCR results showed that *AMBP* knockdown markedly increased the expression of the osteogenic marker genes, *RUNX2* and *OSTERIX*, under OM induction, indicating that AMBP inhibits osteoblastic differentiation (Figure 3B). Consistent results were obtained using western blot analysis (Figure 3C). Alizarin Red S staining further demonstrated that OM significantly induced calcium deposit accumulation and calcified nodule formation. *AMBP* knockdown significantly enhanced OM-induced calcium deposition and nodule formation (Figure 3D). These findings suggest that AMBP knockdown promotes osteoblastic differentiation of VICs and calcium deposition under OM conditions.

In addition, primary VICs were transfected with an *AMBP* overexpression adenovirus to clarify the effect of AMBP on VICs calcification *in vitro*. Successful overexpression of *AMBP* was confirmed by a significant increase in AMBP expression by western blotting, supporting its use in subsequent functional studies (Figure 3E). qRT-PCR results showed that *AMBP* overexpression markedly reduced the expression of the osteogenic markers, *RUNX2* and *OSTERIX*, under OM induction (Figure 3F). Western blotting analysis corroborated these findings (Figure 3G). Alizarin Red S staining further demonstrated that *AMBP* overexpression significantly reduced OM-induced calcium deposition and nodule formation (Figure 3H). These results suggest that *AMBP* overexpression inhibits the differentiation of VICs into an osteoblastic phenotype and plays a crucial role in reducing calcium deposition under OM conditions.

### AMBP knockdown activates the mitogen-activated protein kinase (MAPK) signaling pathway in VICs

**Figure 4:**
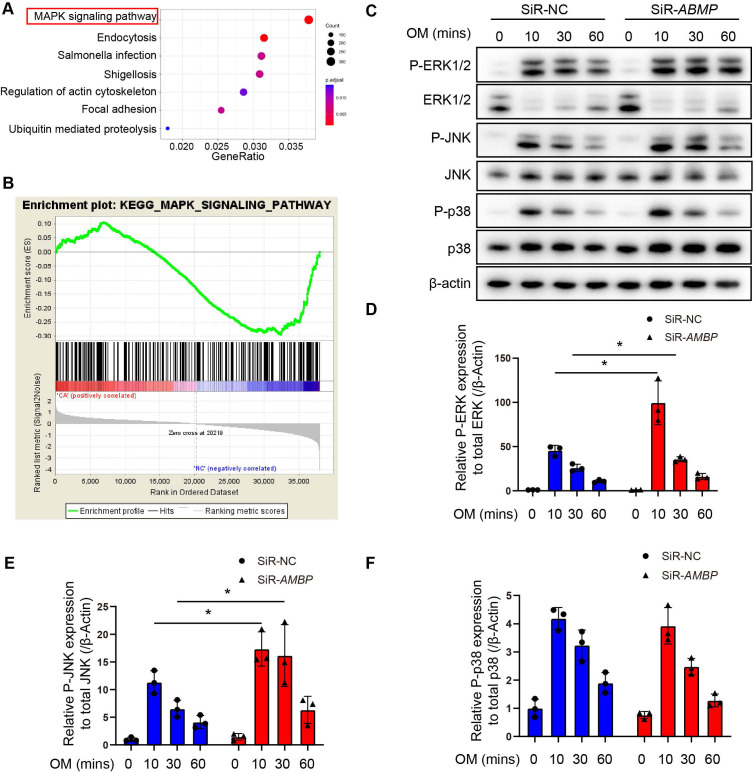
** AMBP knockdown increases phosphorylation of ERK1/2 and JNK. (A-B)** RNA-Seq was conducted on AV tissues from patients with CAVD and non-CAVD controls to identify differentially expressed genes and molecular pathways associated with disease pathogenesis. **(A)** Bubble plot illustrating KEGG pathway analysis of upregulated DEGs. Size of the bubbles represents the number of genes associated with each pathway, while the color gradient indicates the adjusted p-value (p.adjust), with more significant pathways shown in red. The x-axis displays the gene ratio, and the y-axis lists the KEGG pathways. **(B)** Enrichment plot of MAPK signaling pathway from GSEA.** (C)** Representative Western blot images of P-ERK1/2, ERK1/2, P-JNK, JNK, P-p38, and p38 in primary VICs transfected with SiR-*AMBP* or SiR-NC for 24 h following induction with OM at different time points. **(D-F)** Quantitative analysis of the ratio of P-ERK1/2 to total ERK1/2 (**D,** n = 3), P-JNK to total JNK (**E,** n = 3), and P-p38 to total p38 (**F**, n = 3). Values are presented as mean ± SEM. *p < 0.05. Abbreviations: AV, aortic valve; DEG, differentially expressed gene; GSEA, gene set enrichment analysis; KEGG, Kyoto Encyclopedia of Genes and Genomes; MAPK, mitogen-activated protein kinase; OM, osteogenic medium; P-ERK1/2, phosphorylated ERK1/2; P-JNK, phosphorylated JNK; P-p38, phosphorylated p38; RNA-Seq, RNA sequencing; SEM, standard error of the mean; SiR-*AMBP*, small interfering RNA targeting *AMBP*; SiR-NC, small interfering RNA negative control; VICs, valvular interstitial cells.

To investigate the mechanism by which AMBP protects against VICs osteoblastic differentiation and calcification, we performed a KEGG pathway analysis using RNA-seq data from differentially upregulated genes in patients with CAVD compared with controls. The mitogen-activated protein kinase (MAPK) signaling pathway as the most significantly enriched pathway in the CAVD group (Figure 4A). Similarly, previous studies have shown that the MAPK pathway mediates osteoblast differentiation and RUNX2 expression in osteoblast-like cell lines, MG 63 and COS7 26, 27. Gene set enrichment analysis (GSEA) of the MAPK signaling pathway was performed to investigate its role on a genome-wide scale. Based on the GSEA results, the MAPK signaling pathway was significantly enriched in the gene expression data of patients with CAVD (Figure 4B). This indicates a strong association between the MAPK pathway and CAVD development, further supporting its potential role in the pathophysiological mechanisms underlying the disease. Next, we examined the effects of AMBP on the three key members of the MAPK pathway: p38, JNK, and ERK1/2. Our results demonstrate that OM exposure significantly induced the phosphorylation of ERK1/2 (P-ERK1/2), JNK (P-JNK), and p38 (P-p38) in a time-dependent manner. Specifically, P-ERK1/2, P-JNK, and P-p38 levels peaked 10 min after OM treatment and gradually decreased at 30 and 60 min, indicating activation of the MAPK pathway in response to OM stimulation (Figure 4C). Additionally, OM treatment significantly reduced total ERK1/2 levels, increased total p38 levels, and had no effect on total JNK levels (Figure 4C).

*AMBP* knockdown significantly increased the ratio of P-ERK1/2 to total ERK1/2 (Figure 4D) and that of P-JNK to total JNK (Figure 4E), with no significant change in the ratio of P-p38 to total p38 (Figure 4F). Conversely, AMBP overexpression significantly decreased the ratio of P-ERK1/2 to total ERK1/2 and that of P-JNK to total JNK, while no significant change in the ratio of P-p38 to total p38 was observed (Figure S7). These findings indicate that AMBP significantly inhibits the phosphorylation of ERK1/2 and JNK in the MAPK signaling pathway. The regulatory role of AMBP suggests that it impacts osteoblastic differentiation via the MAPK pathway in VICs, indicating that MAPK may be a crucial pathway in this cellular process.

### AMBP promotes proteasome degradation of the activated MAPK pathway via competitively binding to the ZF domain of FHL3

**Figure 5:**
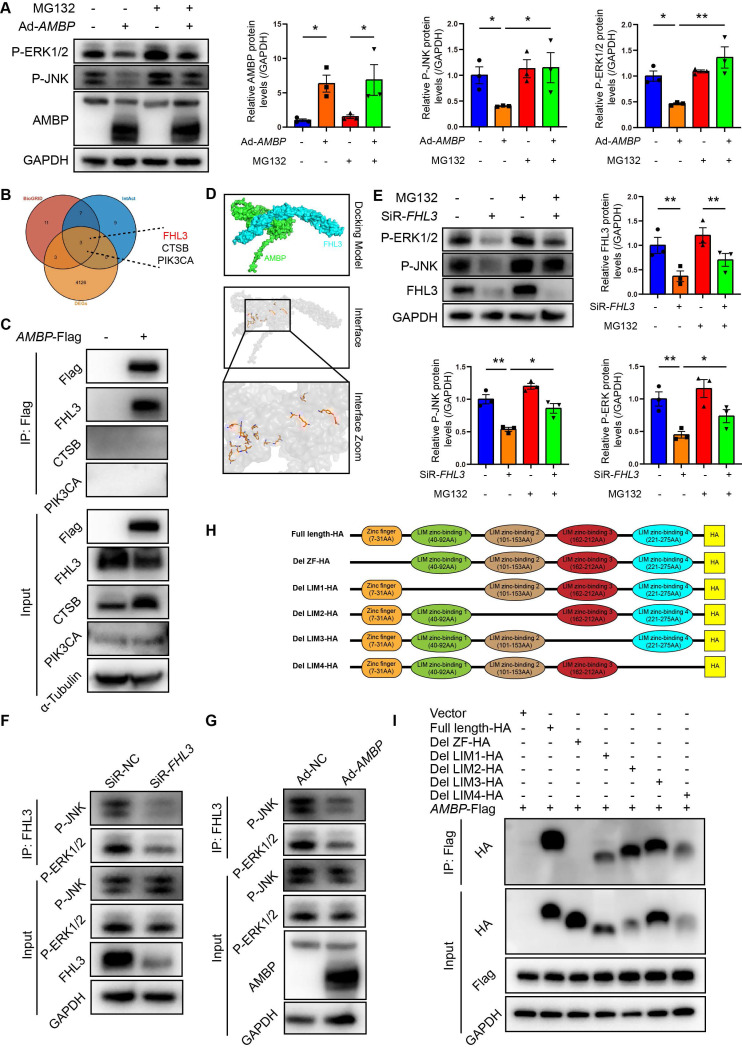
** AMBP promotes proteasome degradation of activated MAPK pathway via competitively binding to ZF domain of FHL3.** (**A**) Representative western blot images and quantification of P-ERK1/2 and P-JNK expression in VICs transfected with Ad-*AMBP* or Ad-NC following pretreatment with ubiquitin-proteasome specific inhibitor, MG132, for 1 h and OM induction (n = 3). (**B**) Venn diagram shows intersection of BioGRID, IntAct, and DEGs derived from this study. (**C**) Co-IP assays confirming interaction between AMBP and FHL3. HEK293T cells are transfected with Flag-tagged *AMBP* overexpressing plasmid for 48 h. Cell lysates are subjected to Co-IP using Flag antibodies, followed by immunoblotting for FHL3, CTSB, and PIK3CA to assess their interaction with AMBP. (**D**) Predicted interaction model between AMBP and FHL3, generated using AlphaFold3. The crystal structure simulation visualized with PyMOL highlights the interaction interfaces between the two proteins. (**E**) Representative western blot images and quantification of P-ERK1/2 and P-JNK expression in VICs transfected with SiR-*FHL3* or SiR-NC following pretreatment with MG132 for 1 h and OM induction (n = 3). (**F**) Co-IP assays confirming interaction between FHL3 and P-JNK or P-ERK1/2 when FHL3 is knocked down. VICs are transfected with SiR-FHL3 or SiR-NC for 48 h, followed by treatment with OM for 10 min. Cell lysates are subjected to Co-IP using FHL3 antibodies, followed by immunoblotting for P-JNK and P-ERK1/2 to assess their interaction with FHL3. (**G**) Co-IP assays were performed to validate the interaction between FHL3 and P-JNK or P-ERK1/2 following AMBP overexpression. VICs are transfected with Ad-*AMBP* or Ad-NC for 48 h, followed by treatment with OM for 10 min. Cell lysates are subjected to Co-IP using FHL3 antibodies, followed by immunoblotting for P-JNK and P-ERK1/2 to assess their interaction with FHL3. (**H**) Schematic representation of primary structural domains of FHL3, highlighting five predicted functional domains: a ZF domain and four LIM zinc-binding domains (LIM1, LIM2, LIM3, and LIM4). (**I**) Co-IP assay demonstrating interaction between AMBP and full-length or truncated forms of FHL3. HEK293T cells are transfected with *AMBP*-Flag and either full-length or truncated FHL3-HA plasmids for 48 h. Cell lysates are subjected to Co-IP using Flag antibodies, followed by immunoblotting with HA antibodies to evaluate the interaction with *AMBP*-Flag. Values are presented as mean ± SEM. *p < 0.05; **p < 0.01. Abbreviations: Ad-NC, negative control adenovirus; Ad-*AMBP*, AMBP overexpressing adenovirus; AMBP-Flag, Flag-tagged AMBP overexpressing plasmid; Co-IP, Co-immunoprecipitation; OM, osteogenic medium; P-ERK1/2, phosphorylated ERK1/2; P-JNK, phosphorylated JNK; P-p38, phosphorylated p38; SEM, standard error of the mean; SiR-*FHL3*, small interfering RNA targeting *FHL3*; SiR-NC, small interfering RNA negative control; VICs, valvular interstitial cells; ZF, zinc finger.

Intracellular protein degradation is a fundamental process for maintaining cellular homeostasis, primarily governed by the ubiquitin-proteasome system (UPS) and autophagy 28, 29. The UPS is responsible for degrading over 80% of intracellular proteins 28. Recent studies have implicated UPS pathway in the osteogenic transdifferentiation of vascular smooth muscle cells 30, 31, with ubiquitination playing a role in ERK1/2 degradation in HEK293T cells 32. Therefore, we aimed to investigate whether AMBP-mediated inhibition of P-JNK and P-ERK1/2 is mediated by the UPS in VICs by employing the proteasome inhibitor, MG132, to block UPS-mediated degradation. Western blot analysis revealed that the inhibitory effect of AMBP on P-JNK and P-ERK1/2 expression was significantly reversed following MG132 pretreatment (Figure 5A), indicating that AMBP suppression by P-JNK and P-ERK1/2 was UPS-mediated. To investigate how AMBP exerts this effect, we used the PPI databases, IntAct and BioGRID, to identify proteins potentially interacting with AMBP and cross-referenced these with DEGs from our study (Figure 5B). We identified three potential AMBP interactors: FHL3, CTSB, and PIK3CA. Co-immunoprecipitation (Co-IP) assays confirmed that AMBP interacted significantly with FHL3, but not with CTSB or PIK3CA in HEK293T cells (Figure 5C). AlphaFold3 was used to simulate the crystal structures and interaction interfaces between AMBP and FHL3 (Figure 5D).

Next, we explored how FHL3 mediates AMBP ubiquitination and degradation of P-JNK and P-ERK1/2. Previous studies have reported that FHL3 knockdown significantly inhibits the phosphorylation of the MAPK pathway components, including p38, ERK1/2, and JNK in gastric cancer cell lines, with FHL3 preventing substrate protein ubiquitination and degradation 33. In this study, we constructed siRNAs targeting *FHL3* and selected the siRNA with the highest knockdown efficiency for subsequent experiments (Figure S8). Western blotting results demonstrated that FHL3 knockdown in OM-induced VICs significantly reduced P-JNK and P-ERK1/2 expression, whereas pretreatment with MG132 reversed this effect (Figure 5E). Co-IP assays further indicated that FHL3 directly bound to P-JNK and P-ERK1/2, and that this interaction was notably diminished following *FHL3* knockdown in OM-induced VICs (Figure 5F). These findings suggest that in OM-activated VICs, FHL3 binds to P-JNK and P-ERK1/2, protecting them from UPS-mediated degradation. We then examined how AMBP, through its interaction with FHL3, mediates the degradation of P-JNK and P-ERK1/2. Co-IP revealed that *AMBP* overexpression significantly reduced FHL3 binding to P-JNK and P-ERK1/2 in OM-induced VICs (Figure 5G). This suggests that AMBP competitively binds to FHL3, thereby promoting the ubiquitination and degradation of P-JNK and P-ERK1/2 in OM-activated VICs. To elucidate the key domains of FHL3 required for its interaction with AMBP, we analyzed the primary structural domains of FHL3 and identified five predicted functional domains: a zinc finger domain (ZF, 7-31 AA) and four LIM zinc-binding domains (LIM1, 40-92 AA; LIM2, 101-153 AA; LIM3, 162-212 AA; and LIM4, 221-275 AA) (Figure 5H). We constructed plasmids encoding full-length FHL3 and five truncated fragments of FHL3 fused with an HA tag at the C-terminus and co-transfected these with a Flag-tagged *AMBP* overexpression plasmid into HEK293T cells. Using anti-Flag antibodies to pull down FHL3-HA, Co-IP results revealed that the deletion of the ZF domain abolished the interaction between FHL3 and AMBP, while full-length FHL3 and truncated fragments lacking LIM1, LIM2, LIM3, and LIM4 domains retained their ability to bind AMBP (Figure 5I). These results suggested that the ZF domain of FHL3 is critical for its interaction with AMBP. In summary, these findings demonstrate that AMBP, by competitively binding to the ZF domain of FHL3, disrupts FHL3's protective effect on P-JNK and P-ERK1/2, preventing their degradation via the UPS. This leads to enhanced ubiquitination and subsequent degradation of P-JNK and P-ERK1/2 in OM-activated VICs. These results reveal a novel regulatory mechanism by which AMBP modulates MAPK signaling in calcific AV disease.

### AMBP protects against osteoblastic differentiation and calcification of VICs through inhibiting the MAPK pathway

We investigated whether P-ERK1/2 and P-JNK mediated the protective effect of AMBP against differentiation of VICs into osteoblasts using specific P-ERK1/2 and P-JNK inhibitors. Western blot analysis revealed that siRNA-mediated *AMBP* knockdown led to significant upregulation of RUNX2 and OSTERIX expression.

**Figure 6:**
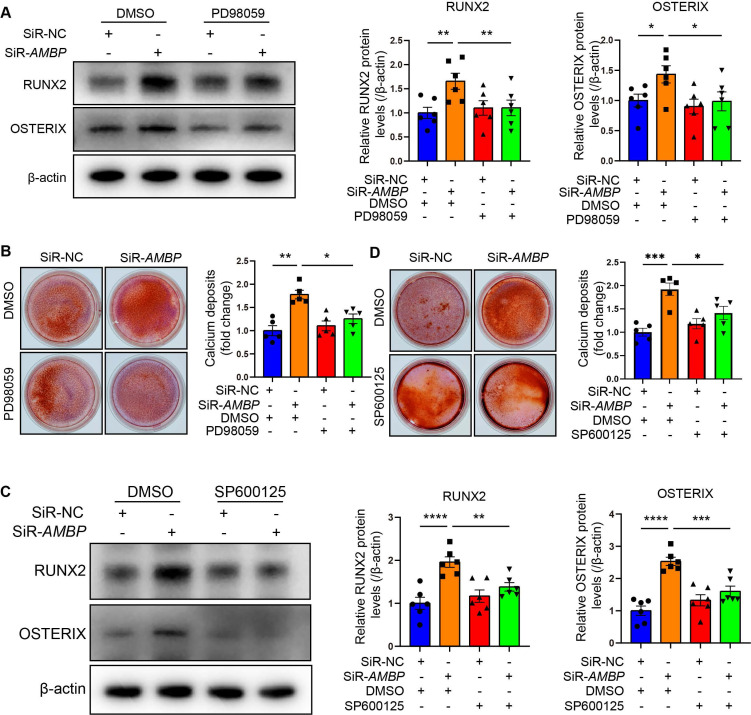
** AMBP protects against osteoblastic differentiation and calcification in VICs through inhibiting MAPK pathway. (A)** Representative western blot images and quantification of RUNX2 and OSTERIX expression in VICs transfected with SiR-*AMBP* or SiR-NC following pretreatment with P-ERK1/2 specific inhibitor, PD98059, for 1 h and OM induction for 72 h (n = 6). **(B)** Alizarin Red S staining and calcium deposit quantification in VICs transfected with SiR-*AMBP* or SiR-NC following pretreatment with PD98059 for 1 h and OM induction for 7 d (n = 5). **(C)** Representative western blot images and quantification of RUNX2 and OSTERIX expression in VICs transfected with SiR-*AMBP* or SiR-NC following pretreatment with P-JNK specific inhibitor, SP600125, for 1 h and OM induction for 72 h (n = 6). **(D)** Alizarin Red S staining and calcium deposit quantification in VICs transfected with SiR-*AMBP* or SiR-NC following pretreatment with SP600125 for 1 h and OM induction for 7 d (n = 5). Values are presented as mean ± SEM. *p < 0.05; **p < 0.01; ***p < 0.001; ****p < 0.0001. Abbreviations: DMSO, dimethyl sulfoxide, as the solvent control of inhibitors; OM, osteogenic medium; SiR-*AMBP*, small interfering RNA targeting *AMBP*; SEM, standard error of the mean; SiR-NC, small interfering RNA negative control; VICs, valvular interstitial cells.

However, pretreatment with the P-ERK1/2 specific inhibitor, PD98059, markedly attenuated this increase (Figure 6A and Figure S9A). Alizarin Red S staining revealed that *AMBP* knockdown significantly increased calcium deposit accumulation, which was significantly reduced by PD98059 pretreatment (Figure 6B). Similarly, the elevated expression of RUNX2 and OSTERIX due to *AMBP* knockdown was significantly inhibited by pretreatment with the P-JNK-specific inhibitor, SP600125 (Figure 6C and Figure S9B). Alizarin Red S staining also showed that the enhanced calcium deposition due to *AMBP* knockdown was significantly reduced by SP600125 pretreatment (Figure 6D). These results suggest that P-ERK1/2 and P-JNK are key mediators of the protective effects of AMBP against osteoblastic differentiation and calcification of VICs.

### AMBP overexpression mitigates AV calcification through the MAPK pathway in ApoE^-/-^ mice

**Figure 7:**
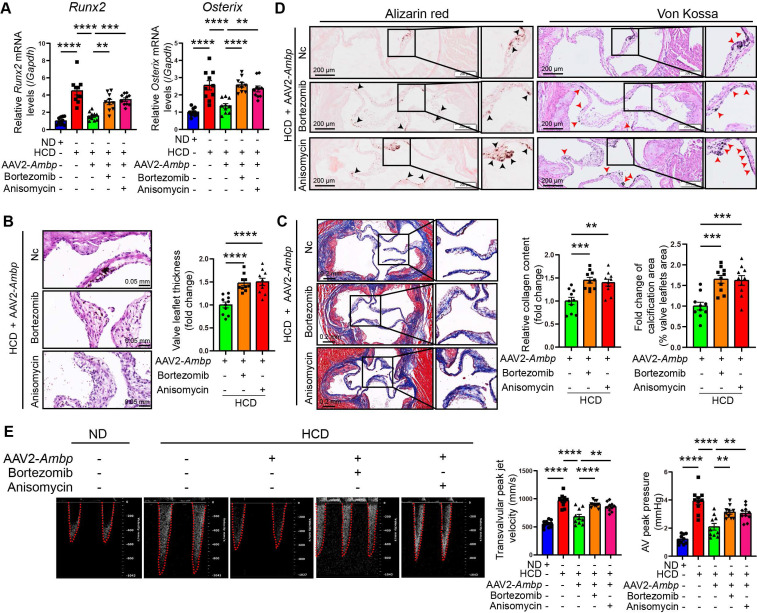
** AMBP overexpression mitigates AV calcification through MAPK pathway in ApoE^-/-^ mice.** (**A-E**) ApoE^-/-^ mice are fed with HCD for 24 weeks after transfecting AAV2-*Ambp* and pretreatment with P-ERK1/2 or P-JNK specific activators, bortezomib or anisomycin, respectively. (**A**) qRT-PCR was used to quantify the mRNA expression levels of *Runx2* and *Osterix* in AVs (n = 10). (**B**) Representative HE images and quantitative analysis of AV thickening in ApoE^-/-^ mice (n = 10, scale bar = 0.05 mm). (**C**) Representative Masson staining images and quantitative analysis of AV fibrosis in ApoE^-/-^ mice (n = 10, scale bar = 0.2 mm). (**D**) Representative Alizarin Red S (black arrows indicate calcified lesions) and Von Kossa (red arrows indicate calcified lesions) staining images and quantitative analysis of AV calcification in ApoE^-/-^ mice (n = 10, scale bar = 200 µm). (**E**) Representative images of pulsed Doppler flow spectrum of AVs and quantitative analysis of transvalvular peak jet velocity and AV peak pressure in echocardiography (n = 10). Values are presented as mean ± SEM. ns, non-significant; *p < 0.05; **p < 0.01; ***p < 0.001; ****p < 0.0001. Abbreviations: AAV2-*Ambp*, *Ambp* overexpressing adeno-associated virus subtype 2; AV, aortic valve; ND, normal diet; HCD, high-cholesterol diet; SEM, standard error of the mean.

To further explore whether AMBP mitigates AV calcification via the MAPK pathway *in vivo*, ApoE^-/-^ mice were injected with *Ambp* overexpressed AAVs via the tail vein and concurrently treated with the ERK1/2 specific agonist, bortezomib, or the JNK agonist, anisomycin, via intraperitoneal injection. The mice were fed a HCD for 24 weeks to induce AV calcification. The results of qRT-PCR in AV tissue indicated that the inhibitory effects of *Ambp* overexpression on the calcification marker genes, *Runx2* and *Osterix*, were significantly diminished following pretreatment with bortezomib and anisomycin (Figure 7A). Histological examination with HE staining revealed that pretreatment with bortezomib and anisomycin significantly reversed the protective effect of AMBP on leaflet thickness (Figure 7B). Similarly, Masson's (Figure 7C), Alizarin Red S, and Von Kossa staining (Figure 7D) demonstrated that the protective effects of AMBP against fibrosis and calcification of the AVs were diminished by pretreatment with each activator. Echocardiography demonstrated that the beneficial impact of AMBP overexpression on transvalvular peak jet velocity and AV peak pressure was abolished following bortezomib and anisomycin pretreatment, except for AV peak pressure in the anisomycin group (Figure 7E). These results indicated that the protective effects of AMBP against AV calcification were mediated through the MAPK pathway in ApoE^-/-^ mice.

## Discussion

In this study, RNA-seq analysis identified seven key hub genes involved in the pathogenesis of CAVD. Among these, *Ambp* emerged as a crucial regulator, significantly mitigating AV calcification in ApoE^-/-^ mice. *In vivo* experiments confirmed that overexpression of *Ambp* markedly reduces AV calcification and leaflet fibrosis, leading to improved valve function. Complementary *in vitro* studies demonstrated that *AMBP* knockdown significantly upregulated the expression of the osteogenic markers, RUNX2 and OSTERIX, along with increased calcium deposition in VICs. Conversely, *AMBP* overexpression had the opposite effect. Our* in vitro* studies revealed that AMBP exerts its protective effects by inhibiting the phosphorylation of the ERK1/2 and JNK pathways, which subsequently decreases the expression of RUNX2 and OSTERIX and reduces calcium deposition in VICs. *In vivo* experiments corroborated these findings, further affirming that AMBP's protective effects against AV calcification in ApoE^-/-^ mice are mediated through the P-ERK1/2 and P-JNK pathways. Mechanistically, AMBP inhibited ERK1/2 and JNK pathway phosphorylation by competitively binding to the ZF domain of FHL3, thereby disrupting the protective effect of FHL3 on P-JNK and P-ERK1/2 and promoting their ubiquitin-proteasome-mediated degradation. These findings provide significant insights into the molecular mechanisms through which AMBP influences CAVD and suggest its potential as a therapeutic target.

Our comprehensive transcriptome sequencing analysis of AV tissues from patients with CAVD provides several pivotal insights. First, our findings confirmed the dysregulation of genes associated with lipid composition and metabolism, corroborating previous studies that underscored the crucial role of lipid metabolism in the initiation and progression of CAVD 34. Specifically, the observed alterations in apoB, apoA1, apo(a), eNOS, and NADPH oxidase levels in calcified AVs substantiate the involvement of dysregulated lipoproteins and oxidative stress in promoting inflammation and immune cell adhesion 34-36. Furthermore, the enrichment of GO terms related to nitric oxide synthase and immune cell processes underscores the active inflammatory nature of CAVD and highlights the significant contribution of immune cells to disease pathogenesis 37, 38. Second, our KEGG pathway enrichment analysis revealed the significant involvement of ECM-receptor interactions and mineral absorption pathways, emphasizing the pivotal role of aberrant ECM remodeling in CAVD 2, 39. ECM components are essential for orchestrating the pathological processes of fibrosis, chronic inflammation, and leaflet calcification 40. These findings reinforce the concept that CAVD is a complex pathophysiological process involving an intricate interplay between VICs, valvular endothelial cells, inflammatory cells, and the ECM. Although hypercholesterolemia is a well-established risk factor for CAVD, several randomized clinical trials have demonstrated the limited efficacy of lipid-lowering therapies, such as statins, in decelerating AV calcification 9, 10. This underscores the significant challenges in the management of CAVD and highlights the need for alternative therapeutic strategies. Consequently, further research is urgently needed to bridge this therapeutic gap and to develop effective interventions for CAVD.

In this study, we identified seven key hub genes associated with CAVD: *AMBP*, *FGG*,* FGA*, *SERPINC1*, *APOA2*, *APOB*, and *ACAN*. Previous studies have demonstrated that *ACAN*, *FGA*, *FGG*, and *SERPINC1* are critical for regulating ECM proteins, thereby contributing to ECM remodeling and degradation, which is a fundamental process in CAVD pathogenesis 34. Additionally, *APOA2* and *APOB* are integral to lipid metabolism and key factors in CAVD progression. A1M, derived from AMBP, plays a role in regulating oxidative stress and inflammation, both of which are pivotal in the development and progression of CAVD. Furthermore, bikunin as the second AMBP product, serves as a structural component of ECM, further emphasizing the multifaceted role of AMBP in maintaining ECM integrity and modulating inflammatory responses 41. These studies underscore the complex interplay between lipid metabolism, ECM remodeling, and inflammatory processes in CAVD. Understanding the specific roles and interactions of these hub genes will provide valuable insights into the molecular mechanisms underlying CAVD and highlight potential therapeutic targets for managing this debilitating condition. Among the identified hub genes, *AMBP* emerged as a potential key regulator of CAVD pathogenesis. Previous studies have shown that AMBP expression is upregulated in response to oxidative stress, indicating its role in antioxidant defense mechanisms 41. Consistent with these observations, our study demonstrated a significant increase in AMBP expression in VICs from the AV tissues of patients with CAVD. Furthermore, our *in vitro* experiments indicated time-dependent upregulation of AMBP expression in VICs following osteogenic stimulation. These findings collectively suggest that AMBP plays a pivotal role in the regulation of CAVD.

However, the role of AMBP in the pathogenesis of CAVD has not been extensively studied. To validate the effects of AMBP on osteoblastic differentiation of VICs, we conducted functional assays using primary VICs. Our results showed that *AMBP* knockdown significantly increased osteoblastic differentiation under osteogenic conditions, confirming the protective effect of AMBP against calcium deposition and calcified nodule formation. We further investigated the involvement of the MAPK pathway in mediating the protective effects of AMBP on osteoblastic differentiation. Previous studies have highlighted the significance of MAPKs in regulating osteoblast differentiation 26, 27. Our findings demonstrated that *AMBP* knockdown increased the phosphorylation of ERK1/2 and JNK, whereas the phosphorylation level of p38 remained unchanged. Using specific inhibitors targeting P-JNK and P-ERK1/2, we established that the protective effect of AMBP on osteoblast differentiation was mediated through these pathways. *In vivo* experiments further supported the protective role of AMBP, as overexpression of *Ambp* in ApoE^-/-^ mice significantly reduced AV calcification and fibrosis and improved valve function. These protective effects were attenuated by specific agonists of the P-ERK1/2 and P-JNK pathways, indicating that AMBP protects AV function through these MAPK pathways. Interestingly, our results diverge from those of previous reports that have implicated the P-p38 MAPK pathway in osteoblast differentiation, emphasizing the critical role of the P-ERK1/2 and P-JNK MAPK pathways, which have not been extensively reported in this context 26, 27. In this study, we uniquely focused on the role of AMBP inhibition on the phosphorylation of ERK1/2 and JNK in VICs.

The UPS is responsible for the degradation of over 80% of intracellular proteins 28. In addition to its essential role in normal physiological processes, the UPS is critical in various pathological conditions 28, 32. In this study, we found that the UPS mediated the inhibitory effects of AMBP on P-JNK and P-ERK1/2. Using PPI databases in conjunction with the DEG set identified in this study, we identified FHL3 as a crucial protein that interacts with P-JNK and P-ERK1/2. Furthermore, our findings demonstrate that FHL3 interacts with P-JNK and P-ERK1/2, thereby inhibiting their UPS-mediated degradation. Co-IP experiments showed that AMBP competitively binds to FHL3, thereby promoting the ubiquitination and degradation of P-JNK and P-ERK1/2 in OM-activated VICs. We further determined that the ZF domain is the key domain of FHL3 required for interaction with AMBP. Notably, in this study, bortezomib, a specific ERK1/2 activator, partially diminished the protective effects of AMBP against CAVD. Given that bortezomib is a therapeutic agent for multiple myeloma, clinicians should be vigilant of the progression of AV calcification in such patients. Although further clinical research is required, AMBP administration may mitigate this exacerbation. This finding highlights a critical issue in the field of cardio-oncology that warrants further investigation into the interplay between cancer treatment and cardiovascular health.

In this study, we provide the first evidence that AMBP functions as a critical regulator of CAVD pathogenesis. Unlike previously reported hub genes involved in ECM remodeling (*ACAN*, *FGA*, *FGG*, and *SERPINC1*) or lipid metabolism (*APOA2* and *APOB*), AMBP uniquely modulates VICs osteogenic differentiation and calcification. Our findings revealed that AMBP was significantly upregulated in calcified AV tissues and in VICs under osteogenic conditions. Functional studies further demonstrated that AMBP overexpression attenuated AV calcification in ApoE^-/-^ mice, whereas AMBP knockdown accelerated VICs osteogenic differentiation and calcium deposition* in vitro*. Mechanistically, we uncovered a novel role of AMBP in inhibiting MAPK-mediated osteogenic differentiation by promoting proteasome degradation of phosphorylated JNK and ERK1/2 via competitive binding to the ZF domain of FHL3. The previously unrecognized interaction between AMBP-FHL3 suggests that AMBP exerts a protective effect against VICs calcification by destabilizing MAPK signaling components. Given the absence of pharmacological interventions for CAVD, these findings highlight AMBP as a potential therapeutic target for mitigating valvular calcification and disease progression. Notably, the translational potential of AMBP extends beyond mechanistic insights. Recent advances in transcatheter aortic valve replacement (TAVR) technology provide a clinically viable platform for localized therapeutic delivery. Building on this paradigm, AMBP-encoding AAV2 vectors or recombinant AMBP protein could be delivered intraprocedurally during TAVR, enabling precise modulation of calcification pathways while minimizing systemic exposure. This approach aligns with ongoing efforts to repurpose interventional devices for targeted molecular therapy, potentially bridging our mechanistic findings to clinical application in CAVD management.

This study had several limitations. First, the functional roles of the other six hub genes identified in this study in the osteoblastic differentiation of VICs remain unexplored. Further investigations are warranted to elucidate the contribution of these genes to the pathogenesis of CAVD. Second, owing to the lack of VICs-specific promoters, we did not use VICs-specific conditional knockout mice in this study. Future studies should aim to validate these findings using conditional AMBP-knockout animal models to provide more precise insights into the roles of genes in CAVD. Finally, we did not measure the concentration of AMBP in the serum of patients, leaving its potential as a biomarker for CAVD occurrence, progression, or severity unclear. Recent research indicates that plasma AMBP may facilitate the distinction of heart failure with preserved ejection fraction with pulmonary hypertension from pulmonary arterial hypertension (PAH) 42. Future studies should assess serum AMBP levels in patients with CAVD to evaluate its potential as a clinical biomarker. These limitations highlight important directions for future research, which will be crucial for advancing our understanding of CAVD and developing effective therapeutic strategies.

## Conclusions

In summary, our study identified AMBP as a critical regulator of CAVD pathogenesis. Through comprehensive RNA-seq analysis and experimental verification, we highlighted the significant role of AMBP in modulating osteoblastic differentiation and calcification of VICs via MAPK signaling pathways, specifically through the inhibition of ERK1/2 and JNK phosphorylation. The *in vivo* overexpression of AMBP demonstrated a protective effect against AV calcification in ApoE^-/-^ mice, further validating its potential therapeutic relevance. Mechanistically, AMBP competitively binds to the ZF domain of FHL3, which destroys the inhibitory effect of FHL3 on the ubiquitin-proteasome degradation of P-JNK and P-ERK1/2, thus increasing the ubiquitination degradation of P-JNK and P-ERK1/2 in OM-activated VICs.

Despite the limitations regarding the exploration of other hub genes, lack of VICs-specific genetic models, and unmeasured serum AMBP levels, our findings provide a strong foundation for future research. These results underscore the potential of AMBP not only as a therapeutic target, but also as a possible biomarker for CAVD, paving the way for novel intervention strategies. Continued research is essential to expand our understanding of the molecular mechanisms underlying CAVD and facilitate the translation of these findings into clinical applications.

## Supplementary Material

Supplementary methods, figures and tables.

## Abbreviations

A1M: α-1-microglobulin
AMBP: α-1-microglobulin/bikunin precursor
AAV: adeno-associated virus
AV: aortic valve
AVS: aortic valve stenosis
CAVD: calcific aortic valve disease
DEG: differentially expressed gene
ECM: extracellular matrix
GSEA: gene set enrichment analysis
HCD: high-cholesterol diet
HE: hematoxylin and eosin
MAPK: mitogen-activated protein kinase
qRT-PCR: quantitative real-time polymerase chain reaction
RNA-seq: RNA sequencing
UPS: ubiquitin-proteasome system
VICs: valvular interstitial cells
ZF: zinc finger
