# Supplementary material for: AMBP protects against aortic valve calcification by inhibiting ERK1/2 and JNK pathways mediated by FHL3
Source: Theranostics. 2025 Mar 18;15(10):4398–415. doi: 10.7150/thno.109182 (PMC11984401; doi:10.7150/thno.109182)
Supplement: Supplementary file 1 — Supplementary methods, figures and tables. [file thnov15p4398s1.pdf]

Supplementary material and methods

Patients and sample acquisition

We collected the aortic valve tissues from patients with CAVD who underwent aortic valve replacement due to severe aortic valve stenosis (AVS) for RNA sequencing. Aortic valve samples from patients with non-calcified aortic valve resection due to heart transplantation (recipient heart) or aortic dissection were collected as the control (non-CAVD). The inclusion criteria for CAVD group were as follows: 50-75 years old; undergoing aortic valve replacement due to severe AVS with significantly vavular calcification. The inclusion criteria for non-CAVD group were as follows: non-calcified aortic valve resection due to heart transplantation (recipient heart) or aortic dissection. The exclusion criteria was patients with rheumatic valvular disease, moderate to severe aortic regurgitation, missing clinical data, renal insufficiency (GFR≤30 ml/min/1.73 m^2), intestinal diseases, autoimmune diseases, and those treated with antibiotics or probiotics for infections within 1 month. CAVD was defined as enhanced ultrasonic echo in the aorta root compared with non-CAVD subjects, valvular thickness ≥3mm, and peak systolic cross-valve flow velocity ≥ 1.5 m/s [1]. The aortic valve samples were collected immediately after section, and were snap-frozen in liquid nitrogen and stored at −80°C within 30 minutes or soaked in 4% paraformaldehyde. All procedures were conducted in accordance with the Declaration of Helsinki. The Institutional Review Board of Qilu Hospital of Shandong University approved this study (Approval number: KYLL-202208-003-1) and all patients provided written informed consent.

RNA extraction

Total RNA was extracted with Trizol reagent (Invitrogen, Carlsbad, CA, USA) following the standard operating protocol provided by the manufacturer. The total RNA

was tested by Agilent Bioanalyzer 2100 (Agilent technologies, Santa Clara, CA, US) to detect the integrity of RNA. Qubit® 3.0 Fluorometer (Life Technologies, CA, USA) and Nanodrop One spectrophotometer (Thermo Fisher Scientific Inc, USA) were used to detect the concentration and purity of total RNA.

Library construction and transcriptome deep sequencing

Paired-end libraries were synthesized by using the Stranded mRNA-seq Lib Prep Kit for lllumina (ABclonal, China) following Preparation Guide. Briefly, The poly-A containing mRNA molecules were purified using poly-T oligo-attached magnetic beads. Following purification, the mRNA is fragmented into small pieces using divalent cations under 94°C for 10 min. The cleaved RNA fragments are copied into first strand cDNA using reverse transcriptase and random primers. This is followed by second strand cDNA synthesis using DNA Polymerase I and RNase H. These cDNA fragments then go through an end repair process, the addition of a single 'A' base, and then ligation of the adapters. The products are then purified and enriched with PCR to create the final cDNA library. Purified libraries were quantified by Qubit® 3.0 Fluorometer (Life Technologies, USA) and validated by Agilent 2100 bioanalyzer (Agilent Technologies, USA) to confirm the insert size and calculate the mole concentration. Cluster was generated by cBot with the library diluted to 10 pM and then were sequenced on the Illumina NovaSeq 6000 (Illumina, USA). The library construction and sequencing were performed by Sinotech Genomics Co., Ltd (Shanghai, China)

Data quality control

Raw reads obtained from original sequencing data contain some sequences with sequencing adaptors or low-quality, so it need to be filtered to obtain clean reads. Fastp

software was used to filter the sequences, and the main purpose of which was to exclude the following sequences: 1. the sequencing primer adaptor sequence contained in reads was removed. 2. reads bases with mass Q below 20 at the 3 'end were removed. 3. reads with sequence length less than 25 were removed; 4. ribosome RNA reads of the sequenced target species were removed [2]. The clean reads were compared to the human reference genome (GRCh38.102) using the Hisat2 software (version 2.0.5) with spliced mapping algorithm [3]. The output SAM (sequencing alignment/map) files were converted to BAM (binary alignment/map) files and sorted using SAMtools (version 1.3.1).

Quantitative analysis and differentially expressed genes (DEGs) screening

Gene abundance was expressed as fragments per kilobase of exon per million reads mapped (FPKM). Stringtie software was used to count the fragment within each gene, and TMM algorithm was used for normalization [4]. False discovery rate (FDR) was used to set the P-value significance threshold in multiple tests. The correlation between samples was calculated with Spearman's correlation and displayed in a correlation diagram of gene expression levels. The FactoMineR package in R was used for principal component analysis (PCA) and clustering to evaluate the inter-group differences and intra-group sample duplications.

Differential expression analysis for mRNA was performed using R package edgeR [5]. Differentially expressed RNAs with |log2(FC)| value >1, q value (FDR adjusted P-value) <0.05 and one group’s mean FPKM >1, considered as significantly modulated, were retained for further analysis. The heatmap and Volcano map were painted with the

R heatmap and ggplot2 package, respectively, to visualize theses DEGs.

Data availability

The RNA-seq data have been deposited in GEO under the accession number GSE235995.

Functional enrichment analyses of DEGs

We performed a Gene Ontology (GO) analysis for biological processes (BP), cellular components (CC), molecular function (MF), and a KEGG (Kyoto Encyclopedia of Genes and Genomes) pathway analysis via Metascape (v3.5, San Diego, CA, USA) [6]. The visualization of the results were achieved by the R ggplot2 package.

Protein–Protein Interaction Network construction and hub gene screening

Protein–Protein Interaction (PPI) Network was constructed with the Search Tool for Retrieval interacting Genes (STRING) database (https://string-db.org/) [7]. An interaction with minimum required interaction score of 0.9 were remained in the PPI network. The network was visualized by Cytoscape software (Version 3.9.1) [8]. Then, based on the PPI network constructed above, significant modules are created using the Molecular Complex Detection (MCODE, Version 2.0.2) plug-in of Cytoscape with the parameter of degree cutoff = 2, node score cutoff =0.2, max depth = 100, and k-score = 2. Next, we used Cytohubba, Cytoscap's plugin, to identify the top 30 hub genes [9]. The top 30 hub genes obtained by 5 different algorithms of Cytohubba, including MCC, DMNC, MNC, Degree, and EPC, were intersection.

Tissue and cells total RNA isolation and real-time PCR analysis

AV tissue (30mg per sample) was put into the homogenate tube (containing

zirconia bead), 1ml TRIzol reagent (Invitrogen, Carlsbad, CA, USA) was added, and the homogenizer was used to homogenate the tissue for 3 times, 15s each time. PrimeScript RT Reagent Kit (Takara Biomedical Technology) was used to reverse transcribe 1 μg of RNA to cDNA for each sample. qRT-PCR amplification was performed using SYBR PCR mix (Roche; Mannheim, Germany) with specific primers (Table S1 in the Supplementary materials) in a Bio-RadCFX96TM Real-Time PCR detection system (Bio-Rad Laboratories, Inc., Hercules, CA, USA). The procedure of real-time PCR amplification were: 10 minutes at 95°C for polymerase activation and 35 consecutive cycles for amplification (30 seconds at 95°C for denaturation, 30 seconds at 60°C for annealing, and 30 seconds at 72°C for extension). The GAPDH RNA was amplified as an internal control. ΔCt method was used to calculate the expression levels, and 2-ΔΔCt method was used for comparisons. Total RNA isolation protocol and real-time PCR analysis from cells were consistent with above.

Isolation of primary VICs, cell culture, and transfection

Primary human VICs were isolated from patients with non-calcified aortic valve, whom undergoing heart transplantation procedures (recipient heart) or aortic dissection, as previously reported [10]. Clinical characteristics of patients for cell isolation were listed in Table S2 in the Supplementary materials. Briefly, the vavular tissue was cut into 1 mm^3 small pieces and digested with D-Hank's solution containing 1 mg/ml Collagenase I (Cat. No. LS004196; Worthington, Lakewood, NJ, USA) at 37 °C for 30 minutes (spinning 50 rpm) to remove endothelial cells. The deposit were further digested with a fresh D-Hank's solution containing 1 mg/ml Collagenase I

(Worthington) for 6h at 37 °C. After repeated aspirations to break up the tissue fragments, the cell suspension was collected and filtered through a 100-μm polypropylene cell strainer (Cat. No 15-1100; Biologix; Jinan, China) to filtrate undigested tissue fragment, and further centrifuge at 1,000 rpm for 5 min to precipitate cells. Isolated primary VICs were resuspended and cultured in Dulbecco's modified Eagle's medium (DMEM; Gibco BRL, Gaithersburg, MD, USA) containing 10% FBS (Cat. No. 10100147C; Thermo Fisher Scientific, Waltham, MA, USA) and 1% penicillin and streptomycin (Cat. No. 10378016; Thermo Fisher Scientific) in a humidified atmosphere with 5% CO2 at 37 °C. Osteogenic medium (OM, α-minimal essential medium containing 0.1% FBS, 50 mg/mL ascorbic acid, 50 ng/mL BMP-2, 5 mmol/L β-glycerophosphate, and 100 nmol/L dexamethasone) were used to stimulate osteogenic differentiation as previously described [11]. Cells between passages 3 to 7 were used for further experiments.

VICs were transfected with siRNA using Lipofectamine RNAi MAX Transfection Reagent (Cat. No. 13778150, Thermo Fisher Scientific) according to the manufacturer's instructions. Commercial synthesis of siRNAs against *AMBP*, FHL3, and the negative control was synthesized by Ribobio (Guangzhou, Guangdong, China). The target sequences for the siRNAs against *AMBP* were as follows: SiR-*ABMP*-1, 5′-CCUGGCUGAAGAAGAUCAUTT-3′; SiR-*ABMP*-2, 5′-GGCAACUGGUAACUGAAGUTT-3′; SiR-*ABMP*-3, 5′-GCAACGGUAACAACUUCGUTT-3′; SiR-*ABMP*-4, 5′-GCAACGGGAACAAGUUCUATT-3′; SiR-Negative Contrl, 5′-

UUCUCCGAACGUGUCACGUTT-3′. The target sequences for the siRNAs against *AMBP* were as follows: SiR-*FHL3*-1, 5′-CAUCGAGAAUGUCUGGUCUTTAGACCAGACAUUCUCGAUGTT-3′; SiR-*FHL3*-2, 5′-CAUCGAGAAUGUCUGGUCUTTAGACCAGACAUUCUCGAUGTT-3′. In the mechanism research, VICs were pretreated with P-ERK (50uM; Cat. No. PD98059; TOPSCIENCE, Shanghai, China), P-JNK (10uM; Cat. No. SP600125; TOPSCIENCE), or proteasome inhibitor MG132 (10 μM; Cat. No. HY-13259; MedChemExpress, Shanghai, China) specific inhibitor for 1h and then transfected with siRNA for 48h, followed by OM induction. In protein interaction experiments, HEK293T cells were transfected with plasmids encoding C-terminal Flag-tagged AMBP and C-terminal HA-tagged FHL3 full-length/truncated mutants (BIOSUN, Jinan, China) using Lipofectamine 3000 (Cat. No. L3000015; Thermo Fisher Scientific), following the manufacturer's protocol. *In vitro* AMBP overexpression experiments, OM induced osteogenic differentiation and calcification of the primary VICs after transfection with the constructed *AMBP* overexpressing adenovirus (BIOSUN; MOI=10).

Protein extraction and western blot analysis.

The primary VICs were lysed in RIPA buffer (Sigma-Aldrich, St Louis, MO, USA) with 1X protease inhibitor cocktail (Cat. No. 04693132001; Roche, Indianapolis, IN, USA). BCA Protein Assay Kit (Thermo Fisher Scientific) was used to quantify the concentration of the extracted protein. The extracted proteins were separated using 4–10% gradient Bis–Tris SDS-Gels (Bio-Rad, Hercules, CA, USA) and then transferred

to nitrocellulose membranes (Millipore, Billerica, MA, USA). The blots were treated with 5% non-fat milk at room temperature (23–27°C) for 1 h and then incubated with primary antibodies against RUNX2 (Cat. No. ab92336; Abcam, Cambridge, MA, USA), OSTERIX (Cat. No. ab209484; Abcam), GAPDH (Cat. No. ab8245; Abcam), ERK (Cat. No. 4695; Cell Signaling Technology, Danvers, MA, USA), P-ERK (Cat. No. 4370; Cell Signaling Technology), JNK (Cat. No. 9252; Cell Signaling Technology), P-JNK (Cat. No. 4668; Cell Signaling Technology), p38 (Cat. No. 8690; Cell Signaling Technology), P-p38 (Cat. No. 4511; Cell Signaling Technology), GAPDH (Cat. No. 5174; Cell Signaling Technology), or β-actin (Cat. No. 3700; Cell Signaling Technology) at 4 °C overnight. The next day, the membranes were washed with Tris-Buffered Saline with Tween 20 (TBST) for 10 minutes three times and then incubated with the horseradish peroxidase (HRP)-conjugated secondary antibody (Cat. No. ab6721 for Rabbit; ab6728 for Mouse; Abcam) at room temperature (23–27°C) for 1 h. Finally, the bands were visualized (AMERSHAM ImageQuant 800, GE Healthcare Bio-Sciences AB, Sweden) using an ECL western blotting detection kit (Millipore, Temecula, CA, USA). The intensity of the bands were quantified using ImageJ (National Institutes of Health, Bethesda, MD, USA). The protein expression levels were normalized to those of GAPDH or β-actin.

Co-immunoprecipitation

Forty-eight hours after plasmid transfection, cells were harvested and lysed in non-denaturing lysis buffer (Cat. No. P0013, Beyotime Biotechnology, Shanghai, China) for 30 minutes. Protein A/G magnetic beads (Cat. No. HYK0202; MedChemExpress) were

incubated with IP-grade antibodies at room temperature for 1 hour, followed by the addition of the cell lysate, which was then incubated overnight at 4°C with rotation. The next day, the beads were collected, and the supernatant was discarded. After four washes with PBS containing 0.5% Tween-20, SDS-PAGE loading buffer was added, and the samples were heated at 95°C for 5 minutes. The collected supernatant was subsequently used for Western blot analysis.

Immunofluorescence and immunohistochemical staining

Human and mice AV tissue sections were dewaxed following by antigen repair (Cat. No. C1034; Solarbio; Beijing, China), and transparent with 0.1% TritonX-100 (Cat. No. GC204003; Servicebio, Wuhan, China) for 10 min in phosphate-buffered saline (PBS). For immunofluorescence staining, the sections were then incubated with 2.5% normal goat serum (Cat. No. G1208; Servicebio) in PBS for 30 min at room temperature (23–27°C) and treated with antibodies against AMBP (Cat. No. ER1803-35; HUABIO; Hangzhou, China) and VIM (Cat. No. 5741; Cell Signaling Technology) overnight at 4 °C. The sections were subsequently washed five minutes for three times with PBS and incubated with Alexa Fluor 594 (Cat. No. ab150120; Abcam, Cambridge, MA, USA), 488 (Cat. No. ab150081; Abcam) secondary antibodies (1:200) for 1 h in the dark at 37 °C. The nuclei were stained with DAPI (Cat. No. ab104139; Abcam). For immunohistochemical staining, following deparaffinization and rehydration, tissue sections were subjected to antigen retrieval using a citrate buffer (pH 6.0) at 95°C for 20 minutes. Endogenous peroxidase activity was quenched by incubating sections in 3% hydrogen peroxide for 10 minutes. After washing with PBS, sections were blocked with

2.5% normal goat serum (Cat. No. G1208; Servicebio) for 30 minutes at room temperature to prevent nonspecific binding. Sections were then incubated with primary antibodies specific for AMBP (Cat. No. ER1803-35; HUABIO; Hangzhou, China) overnight at 4°C. Following primary antibody incubation, sections were washed three times with PBS and incubated with a horseradish peroxidase labeled goat anti-Rabbit IgG (Cat. No. A0208; Beyotime) for 30 minutes at room temperature. After additional washes, the sections were incubated with an DAB substrate (Cat. No. P0202; Beyotime) for 5 minutes, resulting in a brown precipitate at the antigen site. Sections were counterstained with hematoxylin, dehydrated through graded ethanol, cleared in xylene, and mounted with a permanent mounting medium (Permount, Thermo Fisher Scientific, Waltham, MA, USA). The stained sections were examined and imaged using a light microscope (Olympus, Tokyo, Japan). Representative images were randomly selected from each group.

Animal experiments

All procedures involving animals were approved by the Institutional Review Board of Qilu Hospital of Shandong University and complied with the Directive 2010/63/EU of the European Community Council on the protection of animals used for scientific purposes. Six-week-old male ApoE$^{-/-}$ mice (C57BL/6J background, Strain No. T001458) were purchased from GemPharmatech Co., Ltd (Jiangsu, China) and housed in a pathogen-free, temperature-controlled environment with a 12:12 hour light-dark cycle. The mice were injected via the tail vein with adeno-associated virus overexpressing AMBP (2×10^11 vg/mouse; CMV promoter; GenePharma, Shanghai,

China) for 2 weeks. Subsequently, they were fed a 0.2% high-cholesterol diet for 24 weeks to induce aortic valve calcification, as previously described in the literature.[12] For *in vivo* mechanistic experiments, prior to initiating the high-cholesterol diet, mice received tail vein injections of AAV-*Ambp*. Concurrently, the ERK activator Bortezomib (Cat. No. S1013; Selleck, Shanghai, China) was administered intraperitoneally at a dose of 1.0 mg/kg once weekly for two weeks. Additionally, the JNK activator Anisomycin (Cat. No. S7409; Selleck, Shanghai, China) was administered intraperitoneally at a dose of 5 mg/kg every other day for a total of seven doses. At the end of the experiment, mice were euthanized with a lethal dose of sodium pentobarbital (100 mg/kg) via intravenous injection, and aortic valve tissues and blood were collected for analysis. Serum concentrations of Glucose, total cholesterol (TC), triglycerides (TG), high-density lipoprotein cholesterol (HDL-C), and low-density lipoprotein cholesterol (LDL-C) were determined using lipid assay kits from Jiancheng Bioengineering Institute (Nanjing, China).

Echocardiography

At the end of the modeling period, echocardiographic assessment of the aortic valve orifice diameter and aortic valve ejection velocity was performed using a 18-38 MHz phased array transducer (MS400) and the VisualSonic VeVo 2100 Imaging System (Toronto, Canada). Mice were anesthetized with 2% isoflurane inhalation and placed on a heating platform maintained at 37±1°C. Pulsed-wave Doppler was utilized to measure the transvalvular peak jet velocity and AV peak pressure in the apical five-chamber view.

Hematoxylin eosin, Masson, Alizarin red S and Von Kossa staining

The frozen section of mouse aortic valve was prepared by OCT embedding with a thickness of 4μm. For Hematoxylin and Eosin (HE) staining, the frozen sections were rewarmed at room temperature of 25°C for 30min, immersed in pre-cooled acetone at 4°C for 10min, and rinsed in a shaker in PBS (PH 7.2-7.4) for 3 times for 5min each time, stained with hematoxylin (Cat. No. G1004; Servicebio, Wuhan, China) for 5 minutes, rinsed in tap water, differentiated in 1% hydrochloric acid ethanol for 10 seconds, rinsed again, blued in 1% ammonia water for 10 seconds, stained with eosin (Cat. No. G1001; Servicebio, Wuhan, China) for 2 minutes, and then dehydrated, cleared, and mounted. For Masson's trichrome staining, sections were treated with Weigert's iron hematoxylin for 10 minutes, Biebrich scarlet-acid fuchsin solution for 5 minutes, phosphomolybdic acid for 5 minutes, and aniline blue for 5 minutes using a Masson's trichrome stain kit (Cat. No. HT15-1KT; Sigma-Aldrich, St. Louis, USA), followed by dehydration, clearing, and mounting. For Alizarin Red S staining, sections were stained with 2% Alizarin Red S solution (Cat. No. A5533; Sigma-Aldrich, St. Louis, USA) for 30 minutes, then dehydrated, cleared, and mounted. For Von Kossa staining, sections were treated with 5% silver nitrate solution under UV light for 1 hour, rinsed, and then treated with 5% sodium thiosulfate solution for 5 minutes using a Von Kossa stain kit (Cat. No. ab150687; Abcam, Cambridge, UK), followed by dehydration, clearing, and mounting. All stained sections were examined and recorded using a light microscope (Olympus, Tokyo, Japan).

VICs alizarin red S staining

Alizarin red S staining for calcium deposits and calcification nodules was performed as described previously [1]. Briefly, treated primary VICs was washed five minutes for three times with PBS and fixed for 30 min in 4% paraformaldehyde, followed by incubation with 0.2% alizarin red solution (Cat. No. C0148S; Servicebio, Wuhan, China) for 1h. Double distilled water was used to remove excessive dye. Alizarin red S staining was observed and photographed with an inverted microscope (Olympus, Tokyo, Japan). Representative images were randomly selected from each group.

Molecular Modeling and Docking

The protein sequences of AMBP and FHL3 were retrieved from the UniProt database. These sequences were submitted to the AlphaFold server for protein structure prediction and interaction analysis [13]. The predicted interaction models were visualized using PyMOL (PyMOL Molecular Graphics System), where polar interactions between the proteins were highlighted to illustrate key binding sites.

Statistical Analysis

GraphPad Prism 9 software (San Diego, CA, USA) were employed to analyze the data. All data are expressed as mean ± standard error of the mean (SEM) or median with interquartile range. The normality tests was tested using the Shapiro–Wilk normality test. For normally distributed data, an unpaired two-tailed Student's t-test was used to determine statistically significant differences between two groups. One-way analysis of variance (ANOVA) followed by Bonferroni multiple comparisons test (with a mixed model with different numbers of replicates per condition) was performed

to determine the statistical difference between multiple groups with one variable and normal distribution. A two-way ANOVA followed by the Bonferroni multiple comparisons test was used to compare multiple groups with more than one variable. For non-normally distributed data, a nonparametric statistical Kruskal–Wallis test followed by Dunn's post-hoc test was performed for multiple comparisons. The statistical significance was set as $p < 0.05$.

Supplemental tables

Table S1. Specific primer sequences used for qRT-PCR amplification

Gene	Primer Sequence (5′-3′)
AMBP (*Homo sapiens*)	Forward: GGTGGGCAACTGGTAACTGA<br>Reverse: CGCCGTACTGGAAAGTCTCA
ACAN (*Homo sapiens*)	Forward: ACTCTGGGTTTTCGTGACTCT<br>Reverse: ACACTCAGCGAGTTGTCATGG
SERPINC1 (*Homo sapiens*)	Forward: TGAATCCCATGTGCATTTACCG<br>Reverse: TGGTAGCAAAGCGGGAATTGG

APOA2 (*Homo sapiens*)	Forward: CTGTGCTACTCCTCACCATCT Reverse: CTCTCCACACATGGCTCCTTT
APOB (*Homo sapiens*)	Forward: TGCTCCACTCACTTTACCGTC Reverse: TAGCGTCCAGTGTGTACTGAC
FGG (*Homo sapiens*)	Forward: TTATTGTCCAACTACCTGTGGC Reverse: GACTTCAAAGTAGCAGCGTCTAT
FGA (*Homo sapiens*)	Forward: AGACATCAATCTGCCTGCAAA Reverse: AGTGGTCAACGAATGAGAATCC
RUNX2 (*Homo sapiens*)	Forward: GGACGAGGCAAGAGTTTCAC Reverse: GAGGCGGTCAGAGAACAAAC
OSTERIX (*Homo sapiens*)	Forward: GTCTGCAACTGGCTCTTCTG

	Reverse:
	GCAGGCAGGTGAACTTCTTC
GAPDH	Forward:
(*Homo sapiens*)	GCACCGTCAAGGCTGAGAAC
	Reverse:
	TGGTGAAGACGCCAGTGGA
Ambp	Forward:
(*Mus musculus*)	GAGTCCCGGATCTATGGAAAATG
	Reverse:
	TCTTGTCCTTAATGCGGCTCA
Runx2	Forward:
(*Mus musculus*)	GACTGTGGTTACCGTCATGGC
	Reverse:
	ACTTGGTTTTTCATAACAGCGGA
Osterix	Forward:
(*Mus musculus*)	GGAAAGGAGGCACAAAGAAGC
	Reverse:
	CCCCTTAGGCACTAGGAGC
Gapdh	Forward:
(*Mus musculus*)	AGGTCGGTGTGAACGGATTTG
	Reverse:
	GGGGTCGTTGATGGCAACA

Table S2. Clinical characteristics of patients for cell isolation

	Values (n=5)
Age, years	51.20±3.64
Female, %	1 (20%)
SBP, mmHg	132.20 ± 4.64
DBP, mmHg	76.80 ± 2.40
Smoking	2 (40%)
HDL-C, mmol/L	0.89 ± 0.13
LDL-C, mmol/L	2.04 ± 0.40
TG, mmol/L	1.31 ± 0.21
UA, mg/dL	405.2 ± 73.18
CR, umol/L	142.00 ± 38.61
ALT, U/L	34.00 (18.95-536.7)
AST, U/L	52.00 (26.30-1630)
IBIL, umol/L	10.72 ± 4.23
DBIL, umol/L	6.50 (4.25-73.10)
LVEF, %	65.20 ± 1.77

Values are mean ± SEM or median with interquartile range. ALT, alanine aminotransferase; AST, aspartic transaminase; CR, creatinine; DBIL, direct bilirubin; HDL-C, high density lipoprotein-cholesterol; IBIL, indirect bilirubin; LDL-C, low

density lipoprotein cholesterol; LVEF, left ventricular ejection fraction; SEM, standard error of the mean; TG, triglyceride; UA, uric acid.

Table S3. Clinical characteristics of CAVD and non-CAVD cases for aortic valve tissue RNA sequencing

	non-CAVD (n=4)	CAVD (n=5)	p value
Age, years	51.75 ± 4.64	64.80 ± 1.69	0.02
Female, %	1 (25%)	1 (20%)	>0.99
SBP, mmHg	130.80 ± 5.69	133.40 ± 6.62	0.78
DBP, mmHg	77.50 ± 2.96	80.80 ± 4.31	0.57
Smoking	1 (25%)	2 (40%)	>0.99
HDL-C, mmol/L	0.89 ± 0.17	1.05 ± 0.08	0.44
LDL-C, mmol/L	2.01 ± 0.51	2.11 ± 0.38	0.88
TG, mmol/L	1.29 ± 0.27	1.07 ± 0.13	0.46
UA, mg/dL	396.8 ± 93.84	333.6 ± 27.46	0.50
CR, μmol/L	112.00 ± 31.38	79.00 ± 4.83	0.28
ALT, U/L	30.50 (14.93–785.5)	17.00 (12.00–20.50)	0.25
AST, U/L	75.50 (21.48–2396)	19.00 (16.50–23.50)	0.29
IBIL, μmol/L	6.68 ± 1.60	10.20 ± 2.47	0.30
DBIL, μmol/L	5.60 (4.03–10.25)	4.20 (3.10–18.15)	0.73
LVEF, %	65.25 ± 2.29	66.60 ± 2.60	0.72
Peak gradient, mmHg	NA	82.60 ± 14.46	NA
Mean gradient, mmHg	NA	48.20 ± 9.27	NA
Peak velocity, cm/s	NA	446.2 ± 43.61	NA

Values are mean ± SEM or median with interquartile range. ALT, alanine aminotransferase; AST, aspartic transaminase; CAVD, calcific aortic valve disease; CR, creatinine; DBIL, direct bilirubin; DBP, diastolic blood pressure; HDL-C, high-density lipoprotein cholesterol; IBIL, indirect bilirubin; LDL-C, low-density lipoprotein cholesterol; LVEF, left ventricular ejection fraction; NA, not available;

SBP, systolic blood pressure; SEM, standard error of the mean; TG, triglyceride; UA, uric acid.

Table S4. List of the top 50 differentially expressed genes

Gene	P-value	q value	log2FC	UP/DOWN
WIF1	6.28E-13	2.41E-08	-6.21587	DOWN
AC012321.1	2.08E-11	3.97E-07	7.410033	UP
IGSF10	9.31E-11	1.19E-06	-4.7161	DOWN
SORCS1	2.54E-10	2.43E-06	-4.97816	DOWN
SFRP1	1.28E-09	9.82E-06	-4.00904	DOWN
TLCD4	2.75E-09	1.76E-05	-3.6502	DOWN
CACNA1H	3.87E-09	2.12E-05	4.623994	UP
IBSP	5.56E-09	2.66E-05	9.416441	UP
BTG2	1.07E-08	4.56E-05	-3.49406	DOWN
SPOCK3	2.1E-08	8.04E-05	-5.07414	DOWN
STK32A	5.39E-08	0.000178	-3.21504	DOWN
FRRS1L	5.59E-08	0.000178	-4.3068	DOWN
C3	8.48E-08	0.00025	-2.60145	DOWN
STMN2	1.29E-07	0.000328	7.379401	UP
AC122718.1	1.26E-07	0.000328	4.736118	UP
SERPINA1	1.55E-07	0.00037	3.812468	UP
NRCAM	2.12E-07	0.000389	-4.20572	DOWN
ABCA8	2.13E-07	0.000389	-3.12052	DOWN

PLPP3	2.11E-07	0.000389	-2.95327	DOWN
ELOVL2	1.85E-07	0.000389	-3.1333	DOWN
RPS14P4	1.8E-07	0.000389	6.910789	UP
MTCO2P2	2.3E-07	0.0004	3.669596	UP
NAT8L	2.41E-07	0.000402	-4.86483	DOWN
FAM171B	2.62E-07	0.000407	-2.68882	DOWN
PDZRN4	2.67E-07	0.000407	-2.96998	DOWN
NANOS1	2.77E-07	0.000407	-3.10961	DOWN
ITM2A	3.47E-07	0.000475	-2.73851	DOWN
CPAMD8	3.47E-07	0.000475	-3.19036	DOWN
MEGF10	4.2E-07	0.000487	-2.98047	DOWN
FREM1	4.02E-07	0.000487	-2.94504	DOWN
TMEM100	3.97E-07	0.000487	-3.29083	DOWN
HAS2	4.32E-07	0.000487	-2.55098	DOWN
PLEKHN1	4.42E-07	0.000487	4.865109	UP
HMGB1P3	4.13E-07	0.000487	5.786384	UP
AL121821.2	4.58E-07	0.000487	-4.51862	DOWN
MYO10	4.94E-07	0.000511	4.072414	UP
KRT18	5.83E-07	0.000573	3.632981	UP
MTCO3P12	5.69E-07	0.000573	2.853577	UP
TNFRSF19	6.43E-07	0.000615	-2.79828	DOWN
CNTN1	6.77E-07	0.00062	-3.67593	DOWN

NDUFA4L2	6.8E-07	0.00062	3.126181	UP
GPX3	7.22E-07	0.000628	-2.39579	DOWN
GNG2	8.46E-07	0.000704	-2.5809	DOWN
SCN7A	8.89E-07	0.000725	-3.87502	DOWN
PRLR	9.63E-07	0.000748	-3.02067	DOWN
AC090559.1	9.76E-07	0.000748	3.256053	UP
FAM133CP	9.99E-07	0.00075	4.724827	UP
AC009531.1	1.11E-06	0.000819	-4.97967	DOWN
ACADL	1.25E-06	0.000893	-2.73592	DOWN
RP9P	1.26E-06	0.000893	3.707387	UP

Table S5. Details of 17 significant modules obtained by MCODE

Module	Score	Nodes	Edges	Node IDs
1	7	7	21	MT-ND4, MT-CO3, MT-CO1, MT-ND3, MT-ND2, MT-ND1, MT-CO2
2	6.333	7	19	CDC20, AURKA, MKI67, TYMS, BUB1B, TOP2A, AURKB
3	5	5	10	SERPINC1, FGB, FGA, FGG, AMBP
4	4.5	5	9	GPIHBP1, APOC1,

				APOC3, APOB, APOA2
5	4	4	6	IRF6, XAF1, IRF7, ISG15
6	3.333	4	5	ADIPOQ, MED19, NCOA1, CEBPA
7	3	3	3	ACAN, MMP9, MMP3
8	3	3	3	SPON1, ADAMTS1, ADAMTSL2
9	3	3	3	ANK3, ANK2, NRCAM
10	3	3	3	CXCR2, GNA14, GNG2
11	3	3	3	CCNE1,

				CCND2, CDK14
12	3	3	3	CDA, TYMP, TK1
13	3	3	3	MET, LAMC3, LAMA2
14	3	3	3	COL4A4, COL4A3, IBSP
15	3	3	3	HSD3B1, CYP11B2, HSD3B2
16	3	3	3	GFRA1, RET, CDH1
17	2.667	4	4	ADH1A, ALDH2, AOX1, MAOA

MCODE, molecular complex detection.

Table S6. List of top 30 hub genes obtained by five algorithms of Cytohubba

Degree	DMNC	EPC	MCC	MNC
HRAS	MT-CO1	HRAS	MT-CO1	AMBP
SDC1	MT-ND3	SDC1	MT-ND3	MMP9
AMBP	MT-ND2	MMP9	MT-ND2	APOH
ALB	MT-ND1	MMP1	MT-ND1	FGG
CDH1	MT-CO2	CDH1	MT-CO2	APOC3
ACAN	MT-ND4	MMP3	MT-ND4	MT-CO1
MMP9	MT-CO3	ACAN	MT-CO3	MT-ND3
AHSG	MKI67	MMP7	AURKA	MT-ND2
FGG	BUB1B	KITLG	TOP2A	MT-ND1
APOA2	AURKA	MET	AURKB	MT-CO2
SERPINC1	TOP2A	FGFR3	CDC20	MT-ND4
SMARCA4	AURKB	APOH	MKI67	MT-CO3
MMP3	CDC20	RET	BUB1B	MMP1
APOH	TYMS	FGG	AMBP	AHSG
APOB	FGB	AMBP	FGG	FGA
APOC3	FGG	APOA2	FGA	APOA2
AURKA	FGA	GFRA1	SERPINC1	SERPINC1
GNG2	XAF1	ALB	FGB	AURKA
MT-CO1	ISG15	AHSG	TYMS	TOP2A
MT-ND3	GPIHBP1	SERPINC1	APOH	AURKB

MT-ND2	ITGAX	APOB	APOA2	CDC20
MT-ND1	APOB	FGF19	APOC3	MMP3
MT-CO2	SERPINC1	APOC3	APOB	APOB
MT-ND4	AMBP	FGA	AHSG	FGB
MT-CO3	APOA2	TIAM1	HRAS	MKI67
MMP1	IRF6	FGB	MMP9	BUB1B
CEBPA	IRF7	GNG2	MMP1	KITLG
FGA	ACAN	KIT	ACAN	IRF6
SMARCD3	ADH1B	PRSS3P2	SDC1	IRF7
TYMS	APOC1	FLT3	MMP3	ACAN

Supplemental figures and figure legends

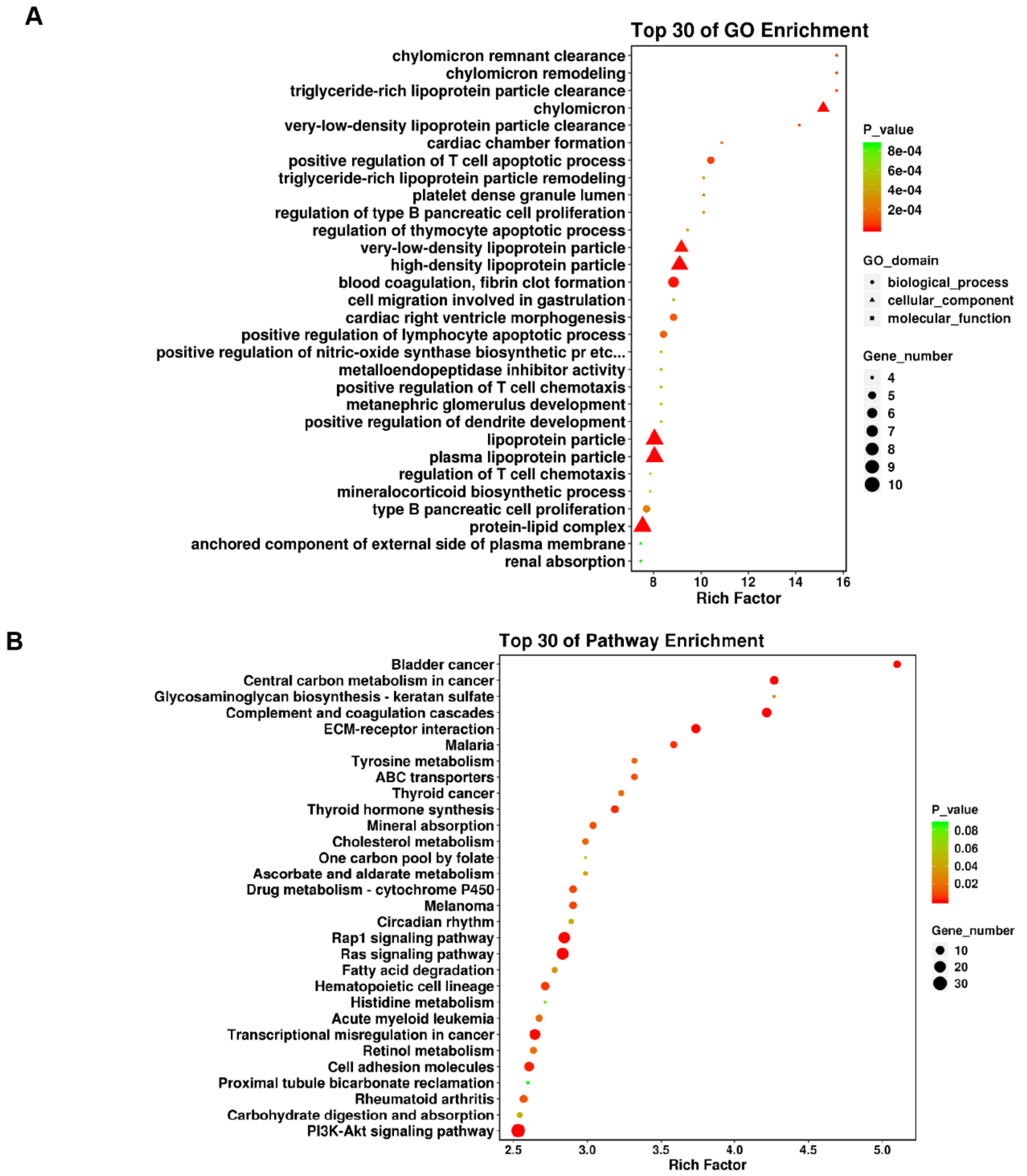

Figure S1. GO and KEGG enrichment analysis of DEGs

(**A**) GO enrichment analysis of DEGs. The top 30 enrichment terms are illustrated; (**B**) KEGG pathway analysis of DEGs. The top 30 enriched pathways are shown. DEGs, differentially expressed genes; GO, Gene Ontology; KEGG, Kyoto Encyclopedia of Genes and Genomes.

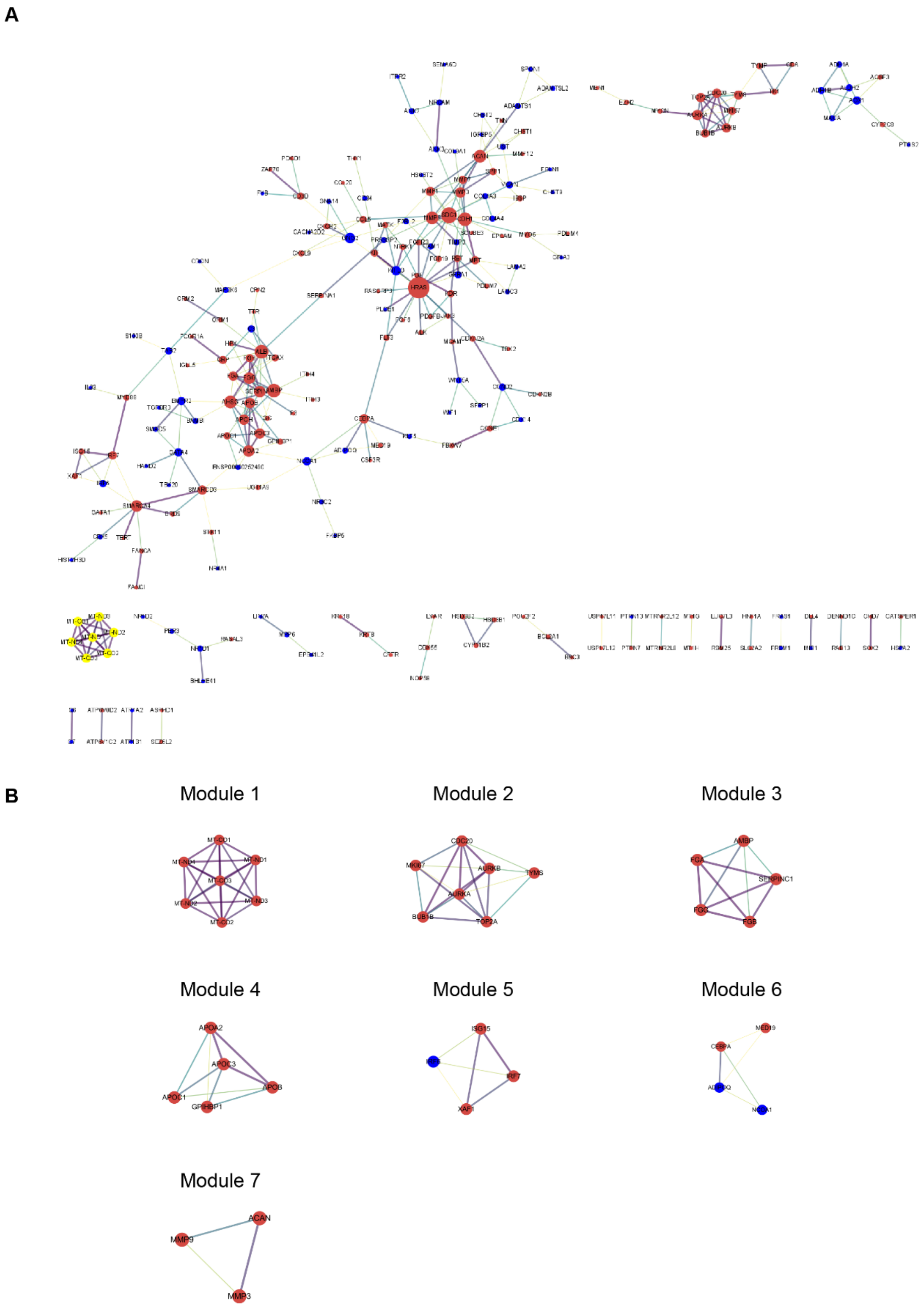

Figure S2. PPI network construction and MCODE analysis.

(A) Constructed PPI network. Genes in red are upregulated; Genes in blue are downregulated; (B) The top seven significant modules were obtained using MCODE. Abbreviations: MCODE, Molecular Complex Detection; PPI, protein-protein interaction.

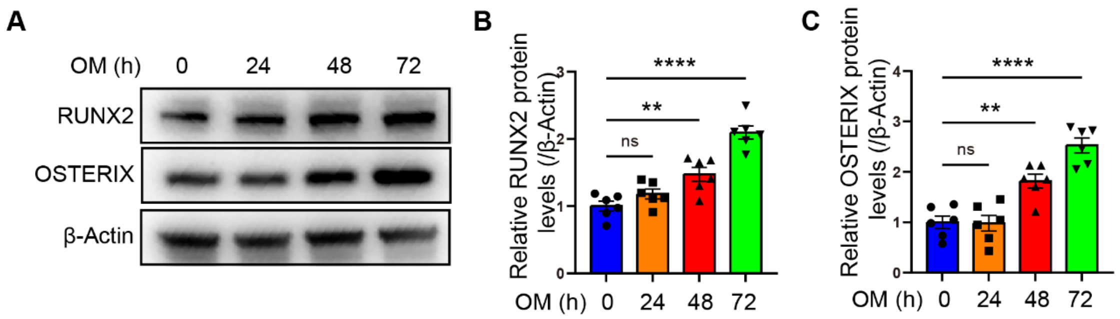

Figure S3. Time-dependent upregulation of osteoblastic differentiation markers by OM in VICs.

(A) Representative Western blot images showing increased RUNX2 and OSTERIX protein expression after OM treatment. (B, C) Quantitative analysis of RUNX2 and OSTERIX expression levels at different time points (n = 6). Data are presented as mean ± SEM. **p<0.01; ****p<0.0001; ns, non-significant. Abbreviations: OM, osteogenic medium.

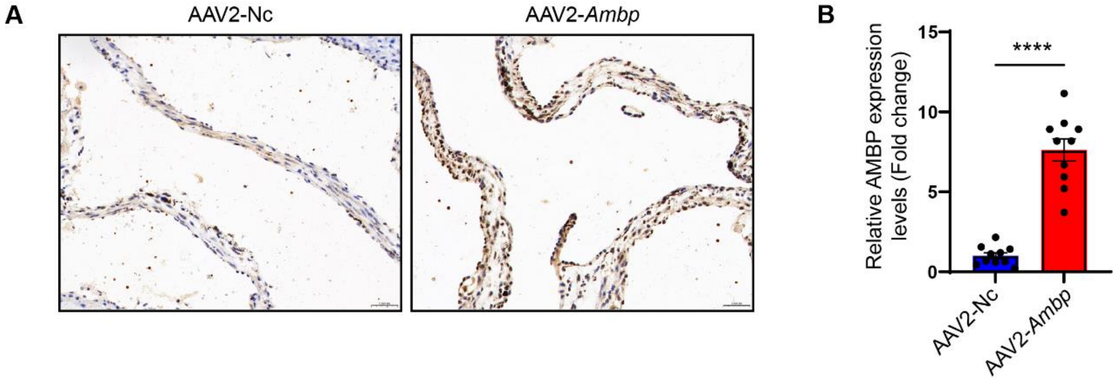

Figure S4. Immunohistochemical analysis of AMBP expression in ApoE$^{-/-}$ mice

aortic valve tissues.

(A) Representative immunohistochemical images showing AMBP expression (brown staining) in aortic valve sections from ApoE$^{-/-}$ mice treated with AAV2-*Ambp* and AAV2-Nc (negative control). Scale bar= 0.05mm. (B) Quantitative analysis of AMBP-positive area (%) in valve tissues (n = 10 mice per group). Data are presented as mean ± SEM. ****p<0.0001. Abbreviations: AAV2-Nc, negative control adeno-associated virus subtype 2; AAV2-*Ambp*, *Ambp* overexpressing adeno-associated virus subtype 2.

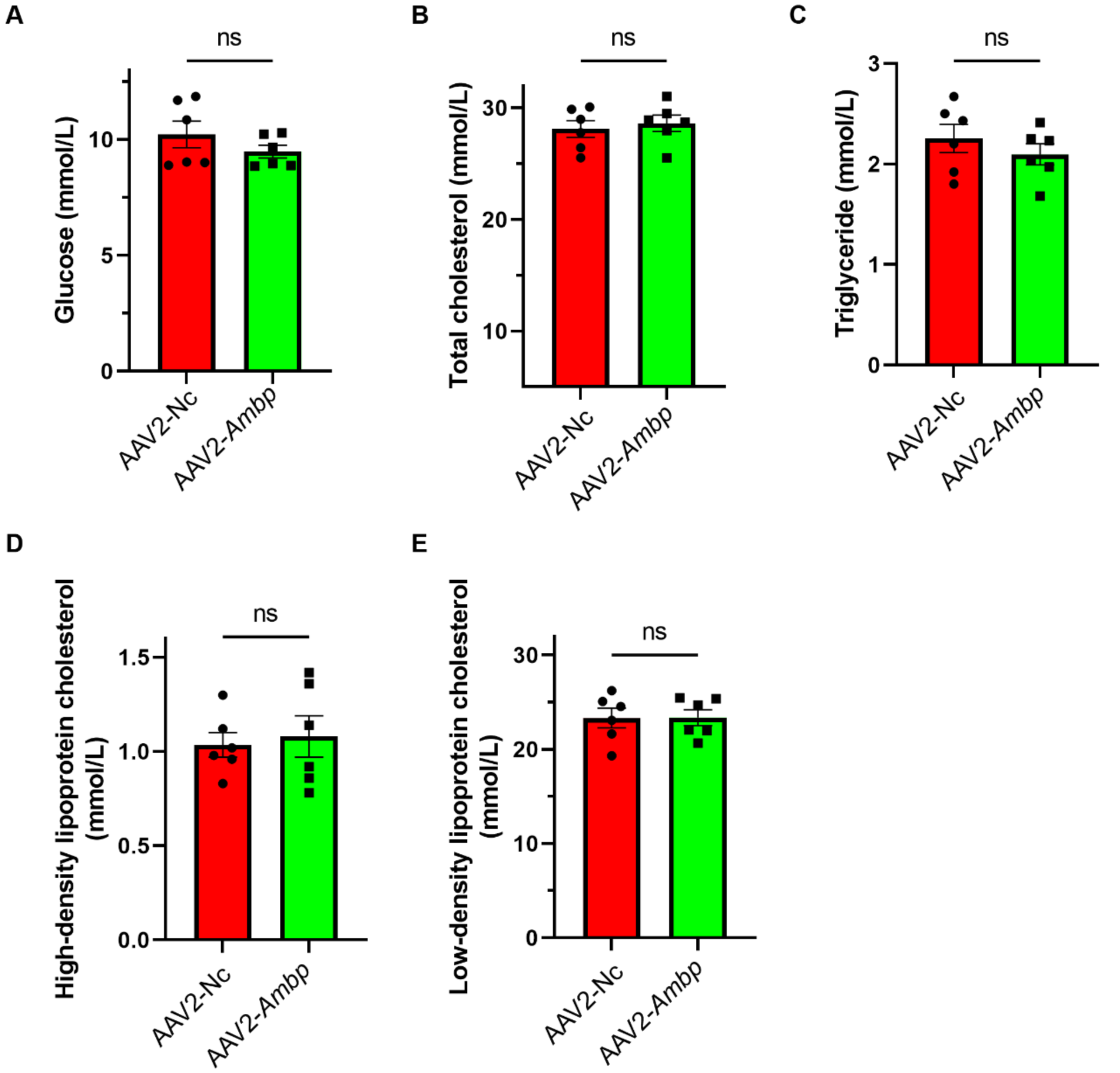

Figure S5. The glucose and blood lipid levels in serum were measured in ApoE$^{-/-}$

mice.

The glucose (A), total cholesterol (B), triglycerides (C), high-density lipoprotein cholesterol (D), and low-density lipoprotein cholesterol (E) were analyzed in serum from ApoE$^{-/-}$ mice fed with HCD for 24 weeks after transfecting AAV2-*Ambp* or AAV2-Nc (n=6). Values are presented as mean ± SEM. ns, non-significant; Abbreviations: AAV2-Nc, negative control adeno-associated virus subtype 2; AAV2-*Ambp*, *Ambp* overexpressing adeno-associated virus subtype 2; HCD, high-cholesterol diet.

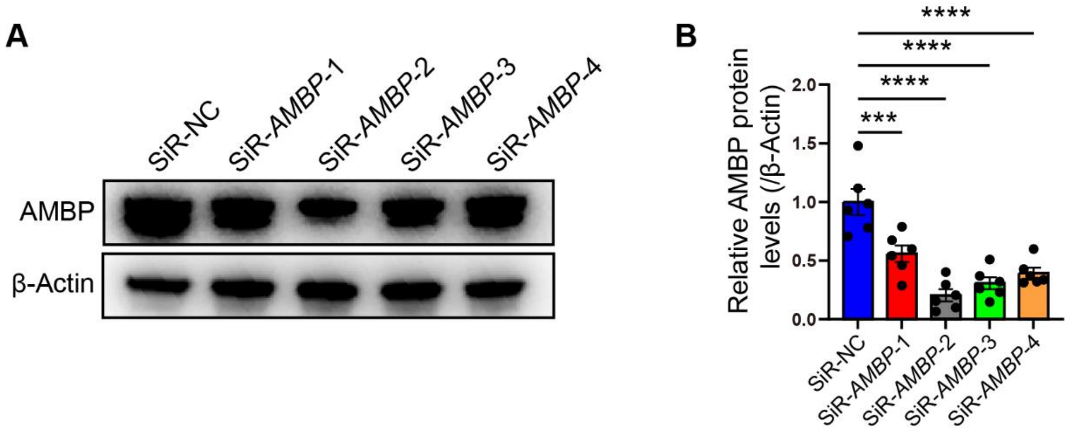

Figure S6. Knockdown efficiency verification of the four siRNAs specifically targeting *AMBP*.

The four siRNAs specifically targeting *AMBP* were designed and transfected into VICs. Representative Western blot images (A) and quantitative analysis (B) were performed to evaluate the efficiency of *AMBP* knockdown (n=6). Values are presented as mean ± SEM. ***p<0.001; ****p<0.0001. Abbreviations: SiR-*AMBP*-1, -2, -3, and -4, No.1, No.2, No.3, and No.4 small interfering RNA targeting *AMBP*; VICs, valvular interstitial cells.

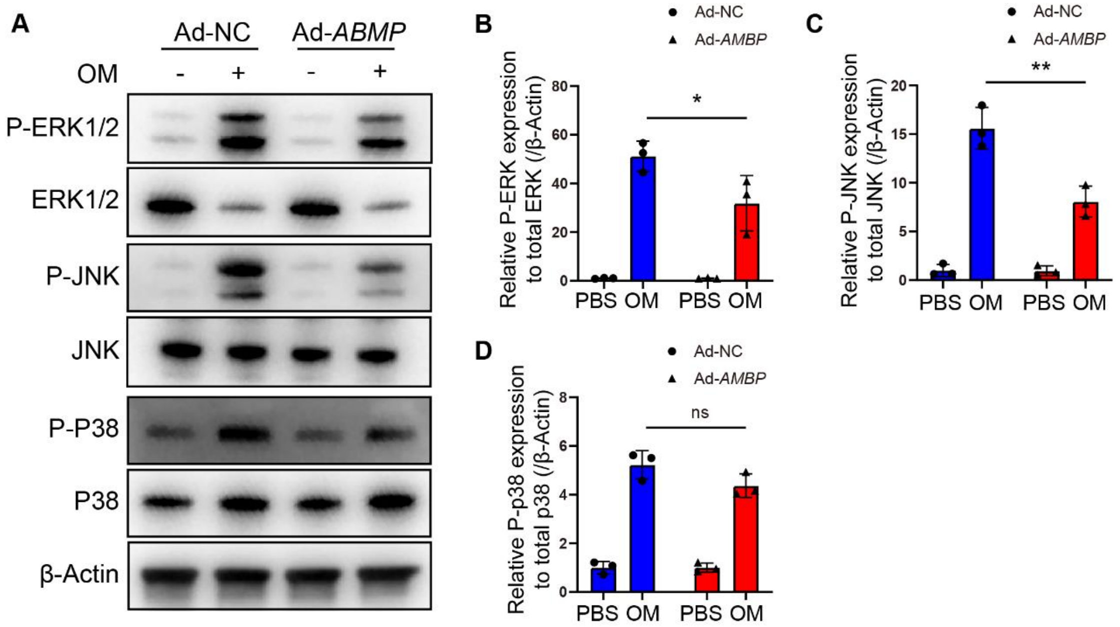

Figure S7. AMBP overexpression inhibits phosphorylation of ERK1/2 and JNK.

(A) Representative Western blot images of P-ERK1/2, ERK1/2, P-JNK, JNK, P-p38, and p38 in primary VICs transfected with Ad-*AMBP* or Ad-NC for 24 h following induction with OM or PBS for 10 minutes. (B-D) Quantitative analysis of the ratio of P-ERK1/2 to total ERK1/2 (B, n=3), P-JNK to total JNK (C, n=3), and P-p38 to total p38 (D, n=3). Values are presented as mean ± SEM. *$p<0.05$; **$p<0.01$; ns, non-significant. Abbreviations: OM, osteogenic medium; PBS, Phosphate buffered saline, as the solvent control; P-ERK1/2, phosphorylated ERK1/2; P-JNK, phosphorylated JNK; P-p38, phosphorylated p38; SEM, standard error of the mean; Ad-*AMBP*, AMBP overexpression adenovirus; Ad-NC, negative control adenovirus; VICs, valvular interstitial cells.

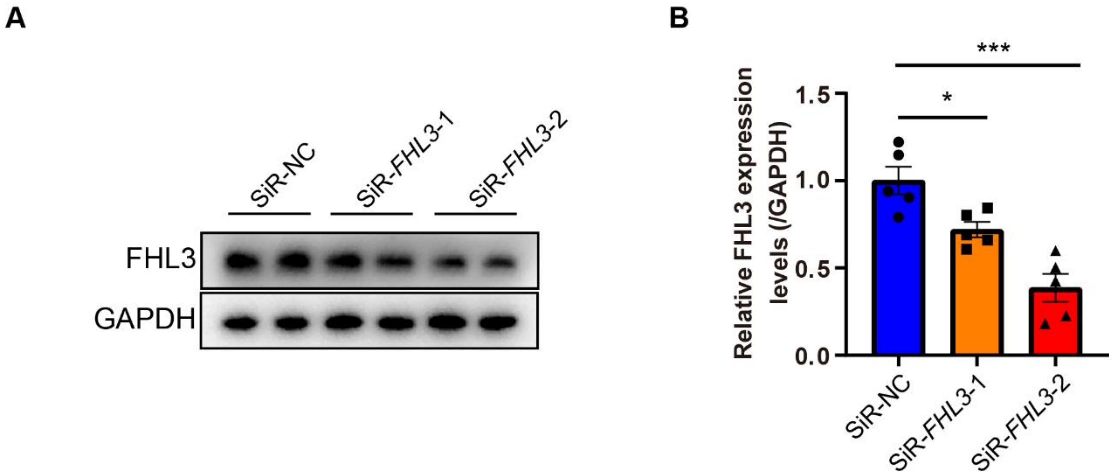

Figure S8. Knockdown efficiency verification of the two siRNAs specifically targeting *FHL3*.

The two siRNAs specifically targeting *FHL3* were designed and transfected into VICs. Representative Western blot images (A) and quantitative analysis (B) were performed to evaluate the efficiency of *FHL3* knockdown (n=5). Values are presented as mean ± SEM. *p<0.05; ***p<0.001. Abbreviations: SiR-*FHL3*-1 and -2, No.1 and No.2 small interfering RNA targeting *FHL3*; VICs, valvular interstitial cells.

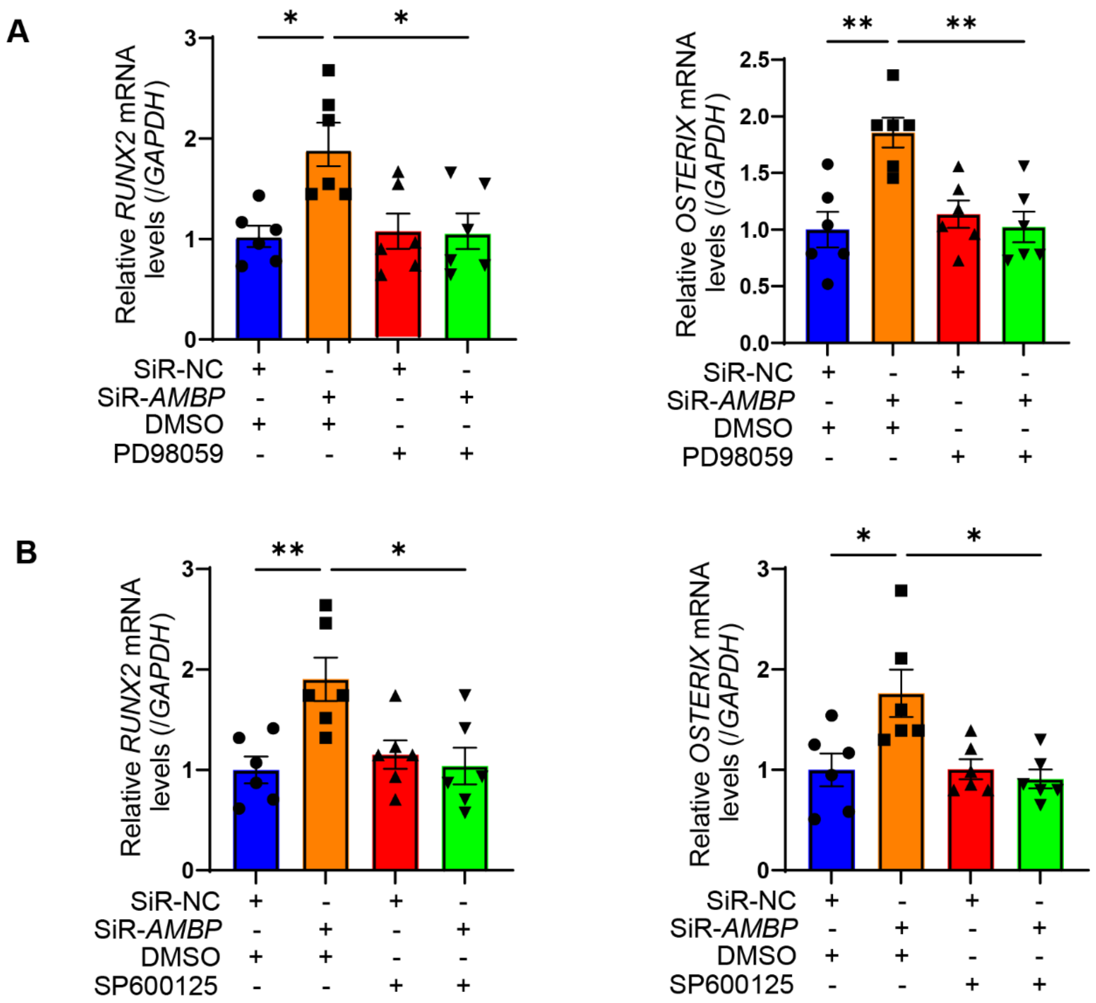

Figure S9. AMBP protects against osteoblastic differentiation and calcification of VICs through inhibiting MAPK pathway.

(A) qRT-PCR was used to quantify the expression of *RUNX2* and *OSTERIX* in VICs transfected with SiR-*AMBP* or SiR-NC following pretreatment with P-ERK1/2 specific inhibitor, PD98059, for 1 h and OM induction for 72 h (n=6). (B) qRT-PCR was used to quantify the expression of *RUNX2* and *OSTERIX* in VICs transfected with SiR-*AMBP* or SiR-NC following pretreatment with P-JNK specific inhibitor, SP600125, for 1 h and OM induction for 72 h (n=6). Values are presented as mean ± SEM. *$p<0.05$; **$p<0.01$. Abbreviations: DMSO, dimethyl sulfoxide, as the solvent control of inhibitors; OM, osteogenic medium; SiR-*AMBP*, small interfering RNA targeting

AMBP; SEM, standard error of the mean; SiR-NC, negative control small interfering RNA; VICs, valvular interstitial cells.

References

1. Li J, Zeng Q, Xiong Z, Xian G, Liu Z, Zhan Q, et al. Trimethylamine N-oxide induces osteogenic responses in human aortic valve interstitial cells in vitro and aggravates aortic valve lesions in mice. Cardiovasc Res. 2022; 118: 2018-30.
2. Chen S, Zhou Y, Chen Y, Gu J. fastp: an ultra-fast all-in-one FASTQ preprocessor. Bioinformatics. 2018; 34: i884-i90.
3. Buchfink B, Xie C, Huson DH. Fast and sensitive protein alignment using DIAMOND. Nat Methods. 2015; 12: 59-60.
4. Zhao Y, Li MC, Konaté MM, Chen L, Das B, Karlovich C, et al. TPM, FPKM, or Normalized Counts? A Comparative Study of Quantification Measures for the Analysis of RNA-seq Data from the NCI Patient-Derived Models Repository. J Transl Med. 2021; 19: 269.
5. Robinson MD, McCarthy DJ, Smyth GK. edgeR: a Bioconductor package for differential expression analysis of digital gene expression data. Bioinformatics. 2010; 26: 139-40.
6. Zhou Y, Zhou B, Pache L, Chang M, Khodabakhshi AH, Tanaseichuk O, et al. Metascape provides a biologist-oriented resource for the analysis of systems-level datasets. Nat Commun. 2019; 10: 1523.
7. Szklarczyk D, Gable AL, Lyon D, Junge A, Wyder S, Huerta-Cepas J, et al. STRING v11: protein-protein association networks with increased coverage, supporting functional discovery in genome-wide experimental datasets. Nucleic Acids Res. 2019; 47: D607-d13.
8. Shannon P, Markiel A, Ozier O, Baliga NS, Wang JT, Ramage D, et al. Cytoscape: a software environment for integrated models of biomolecular interaction networks. Genome Res. 2003; 13: 2498-504.
9. Chin CH, Chen SH, Wu HH, Ho CW, Ko MT, Lin CY. cytoHubba: identifying hub objects and sub-networks from complex interactome. BMC Syst Biol. 2014; 8 Suppl 4: S11.
10. Yu C, Li L, Xie F, Guo S, Liu F, Dong N, et al. LncRNA TUG1 sponges miR-204-5p to promote osteoblast differentiation through upregulating Runx2 in aortic valve calcification. Cardiovasc Res. 2018; 114: 168-79.
11. Xiao X, Zhou T, Guo S, Guo C, Zhang Q, Dong N, et al. LncRNA MALAT1 sponges miR-204 to promote osteoblast differentiation of human aortic valve interstitial cells through up-regulating Smad4. Int J Cardiol. 2017; 243: 404-12.
12. Rajamannan NM. The role of Lrp5/6 in cardiac valve disease: experimental hypercholesterolemia in the ApoE-/- /Lrp5-/- mice. J Cell Biochem. 2011; 112: 2987-91.
13. Jumper J, Evans R, Pritzel A, Green T, Figurnov M, Ronneberger O, et al. Highly accurate protein structure prediction with AlphaFold. Nature. 2021; 596: 583-9.
